# Metabolism of tissue macrophages in homeostasis and pathology

**DOI:** 10.1038/s41423-021-00791-9

**Published:** 2021-12-07

**Authors:** Stefanie K. Wculek, Gillian Dunphy, Ignacio Heras-Murillo, Annalaura Mastrangelo, David Sancho

**Affiliations:** Immunobiology Laboratory, Centro Nacional de Investigaciones Cardiovasculares (CNIC), Melchor Fernández Almagro 3, Madrid, 28029 Spain

**Keywords:** Tissue macrophages, metabolism, homeostasis, pathology, tissue regeneration, Monocytes and macrophages, Innate immunity, Inflammation

## Abstract

Cellular metabolism orchestrates the intricate use of tissue fuels for catabolism and anabolism to generate cellular energy and structural components. The emerging field of immunometabolism highlights the importance of cellular metabolism for the maintenance and activities of immune cells. Macrophages are embryo- or adult bone marrow-derived leukocytes that are key for healthy tissue homeostasis but can also contribute to pathologies such as metabolic syndrome, atherosclerosis, fibrosis or cancer. Macrophage metabolism has largely been studied in vitro. However, different organs contain diverse macrophage populations that specialize in distinct and often tissue-specific functions. This context specificity creates diverging metabolic challenges for tissue macrophage populations to fulfill their homeostatic roles in their particular microenvironment and conditions their response in pathological conditions. Here, we outline current knowledge on the metabolic requirements and adaptations of macrophages located in tissues during homeostasis and selected diseases.

## Introduction

Macrophages are tissue-resident immune cells that act as important immune sentinels and concomitantly execute vital homeostatic tasks to ensure tissue integrity and functionality. Indeed, macrophages are present in virtually every organ of the body. They colonize tissues to form self-maintaining populations, can be replenished by circulating monocytes following insults or are constantly differentiating from infiltrating monocytes. However, the compositions and requirements of different microenvironments and niches differ notably, demanding distinct functions from their resident macrophages [1]. Seminal studies have shown that the identity of macrophage populations is imprinted by the residing tissue, which is evidenced by the expression of tissue-associated signature transcription factors [2–6]. Indeed, after loss of resident macrophages, incoming monocytes are reprogrammed by environmental macrophage niches to adopt the transcriptional programs that define the resident population [7–9]. These mostly tissue-specific transcriptional programs are vital for the functions, maintenance and phenotypes of tissue macrophages. Interestingly, several of these signature macrophage transcription factors regulate fundamental metabolic features [2–6].

In vitro studies in the context of pro- or anti-inflammatory activation highlight the metabolic plasticity of macrophages, which can completely rewire aspects of their cellular metabolism—bioenergetics, nutrient usage, generation of metabolites/cellular building blocks, etc.—depending on the task at hand [10, 11]. These observations suggest that tissue-resident macrophage identity requires a certain metabolic state that may depend on the availability of metabolites or nutrients and, importantly, facilitates their tissue-specific function. Moreover, disease can change the tissue microenvironment and consequently affect macrophage metabolism and function; in turn, macrophage metabolism may be key for disease resolution or progression.

Cellular metabolism is a complex network that is essential for cellular fitness and consists of catabolic processes (degradation of nutrients, predominantly for metabolite or energy generation in mitochondria) and anabolic processes (use of metabolites for synthesis of cellular structures). In short, tissue fuels (such as glucose, lipids or amino acids) are converted into metabolites (such as pyruvate, tricarboxylic acid [TCA] cycle intermediates, fatty acids or free cholesterol) by several metabolic reactions (such as glycolysis, lipolysis or glutaminolysis). These cellular metabolites are either fully oxidized in mitochondria (for example, via the TCA cycle, fatty acid oxidation [FAO] and oxidative phosphorylation [OXPHOS] by the electron transport chain [ETC]), used for the production of cellular building blocks (for example, in amino acid, nucleotide or fatty acid synthesis [FAS]) or released from the cells to prevent toxicity (for example, lactate or excess cholesterol).

Here, we discuss current knowledge on the cellular metabolism of diverse macrophage populations that reside in different tissues, how it supports their homeostatic activities and contributes to defining their identities. We focus on macrophages that have been studied in vivo with similar or context-specific metabolic needs, such as populations in the lung, spleen, liver, peritoneum, brain and bone. In addition, while the metabolism of tissue macrophages during inflammation has been recently reviewed [12], we explain the metabolic and functional adaptations of these cells in selected examples of different types of chronic pathologies in which macrophages are key for disease progression.

## The in vitro paradigm of macrophage metabolism

**Fig. 1:**
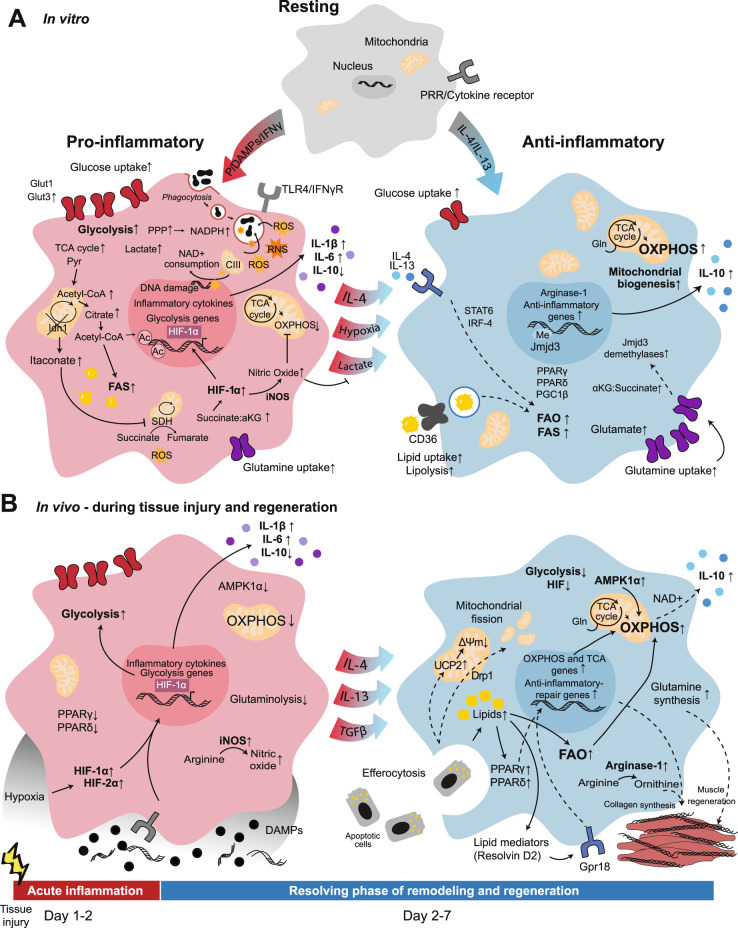
Metabolic rearrangement in macrophage polarization to proinflammatory or alternatively activated macrophages in vitro and in vivo. **A** In vitro, resting macrophages can be activated by various pathogen- or danger-associated molecular patterns (PAMPs or DAMPs), and cytokines polarize to classically activated proinflammatory M1 macrophages or alternatively activated anti-inflammatory M2 macrophages. **B** Upon in vivo tissue injury, damaged cell debris is released into the extracellular microenvironment, and an inflammatory response is mounted. Next, upon clearance of cell debris and DAMPs, the response changes to promote resolution of inflammation. Both in vitro (**A** left) and in vivo (**B** left), proinflammatory polarization has been associated with enhanced glycolytic metabolism; however, the majority of the related information has been elucidated in vitro. Both in vitro (**A** right) and in vivo (**B** right), increases in OXPHOS, FAO and glutaminolysis are associated with alternatively activated macrophages. Ac, Acetylation; CIII, complex III; Drp1, dynamin-related protein 1; Gln, Glutamine; Gpr18, G protein-coupled receptor 18; Me, Methylation; PRR, pattern recognition receptor; Pyr, Pyruvate; UCP2, mitochondrial uncoupling protein 2; ΔΨm, mitochondrial membrane potential. Solid lines: direct relationships; dashed lines: indirect relationships. Black circles: DAMPs; blue circles: anti-inflammatory cytokines; purple circles: proinflammatory cytokines; orange stars: ROS and RNS; black irregular ovals: phagocytosed particles

A large body of literature has focused on macrophage metabolism in vitro [11, 13]. In these studies, resting macrophage colony stimulating factor (M-CSF)-induced bone marrow-derived macrophages (BMDMs) are referred to as M0 macrophages and undergo stimuli-specific differentiation into a range of polarization states, simplistically summarized as M1 at one extreme and M2 at the other [14–16] (Fig. 1A).

Classically activated “proinflammatory” (M1) macrophages are generated via Toll-like receptor (TLR) stimulation with agonists such as lipopolysaccharide (LPS) and/or cytokines such as interferon (IFN)γ [17, 18]. M1 macrophages are characterized by glycolytic metabolism, inducible nitric oxide synthase (iNOS) expression, and the production of proinflammatory cytokines [14, 19] (Fig. 1A). Upon TLR ligation, glucose is taken up, and glycolysis increases [16, 20, 21]. Elevated amounts of glycolytic intermediates promote the pentose phosphate pathway (PPP), which supports nicotinamide adenine dinucleotide phosphate (NADPH) generation for nucleotide biosynthesis and reactive oxygen species (ROS) production [19, 22]. Increased glycolysis leads to enhancement of lactate production and entry of glucose-derived pyruvate into the TCA cycle [23, 24]. This is accompanied by two breaks within the TCA cycle [24, 25]. Isocitrate dehydrogenase (IDH)-1, the enzyme that converts isocitrate to α-ketoglutarate (αKG), is downregulated, allowing accumulation of citrate and synthesis of itaconate [23, 25–27]. Citrate is converted into acetyl-coenzyme A (CoA), which is used first for de novo histone acetylation and inflammatory gene transcription and second as a substrate in FAS, leading to fatty acid accumulation required for expansion of cell membranes and increased protein synthesis [19, 24, 28–31]. Itaconate is one of the most highly upregulated metabolites upon M1 macrophage activation [32–35]. Itaconate inhibits succinate dehydrogenase (SDH; complex II of the ETC), inhibiting the conversion of succinate to fumarate, leading to the second break in the TCA cycle and succinate accumulation [25, 34–37]. Succinate accumulation stabilizes hypoxia-inducible factor (HIF)-1α, promoting a second wave of sustained metabolic reprogramming via gene expression programs [19, 38]. This second phase is characterized by a decrease in mitochondrial respiration and a dependence on glycolysis, similar to the “Warburg effect” described in cancer cells [19, 39]. Itaconate has also been shown to modulate macrophage cytokine production independently of succinate accumulation [40]. Succinate oxidation by SDH promotes proinflammatory responses in LPS-treated cells and induces mitochondrial ROS (mtROS) generation [26, 37, 41]. M1-induced mtROS are recruited to the phagosome to enhance bacterial killing, but they also induce oxidative DNA damage, activating NAD-consuming poly-(ADP-ribose) polymerase (PARP) enzymes and leading to M1 macrophage reliance on the NAD^+^ salvage pathway [42–44]. M1-induced nitric oxide (NO) is antimicrobial but also inhibits the ETC to reduce mitochondrial respiration [27, 45, 46]. Mitochondrial dysfunction due to NO decreases the adenosine triphosphate (ATP):adenosine diphosphate (ADP) ratio in cells, which has been shown to dampen inflammatory responses [47]. However, NO can also prevent M2 repolarization of inflammatory macrophages, potentially impeding the transition to the resolution phase of the immune response [46–48].

Alternatively activated “anti-inflammatory” (M2) macrophages differentiate in response to interleukin (IL)-4 or IL-13 and are characterized by increased mitochondrial respiration, anti-inflammatory cytokine production, and high arginase-1 expression [18, 49, 50] (Fig. 1A). M2 macrophages promote T helper cell (Th)2 responses and the resolution of inflammation [51]. Increased arginase expression in M2 macrophages leads to increases in the activity of ornithine and polyamine biosynthesis pathways. The polyamine spermidine has been shown to hypusinate translation factor eukaryotic initiation factor 5 A, facilitating the expression of mitochondrial proteins required for OXPHOS-dependent M2 differentiation [52]. The energetic profile of these cells is characterized by increased expression of genes related to fatty acid uptake, transport, and oxidation and increased uptake of both glucose and fatty acids in culture [51]. While M1 macrophages increase glycolysis within a very short time frame, M2 macrophages do so only at later time points and do not rely on glycolysis for their differentiation [53, 54]. Instead, M2 macrophages utilize glutamine and FAO to support their metabolic demands upon IL-4 sensing [53, 55]. One-third of TCA carbons are glutamine-derived in M2 cells, whereas one-fifth are glutamine-derived in M1 macrophages [25, 56]. Endocytosis of triacylglycerol-containing lipoproteins is driven by increased CD36 expression on M2 macrophages, dependent on the transcription factors signal transducer and activator of transcription (STAT)6, peroxisome proliferator-activated receptor gamma (PPAR)γ, PPARδ, PGC-1β (a PPAR coactivator), and interferon regulatory factor (IRF)4 [51, 54, 55, 57, 58] (Fig. 1A). An increase in both the uptake of exogenous lipids and FAS supports enhanced FAO and mitochondrial biogenesis, resulting in a higher oxygen consumption rate (OCR) in M2 macrophages than in M0 and M1 macrophages [55]. However, several studies using FAO inhibitors or genetic models that prevent FAO have shown no reliance on FAO for M2 polarization, making it unclear whether the metabolic changes observed in M2 macrophages are responsible for immune polarization or if they are a consequence [46, 59, 60]. Glutaminolysis promotes αKG accumulation, leading to activation of Jumonji domain-containing protein D3 (JMJD3) demethylases [56]. JMJD3-dependent histone demethylation on M2-specific gene promoters is responsible for M2 polarization [25, 56, 61, 62]. IL-4 signaling also activates Akt, which is responsible for increased de novo histone acetylation at M2-specific genes [63]. Whether this activation depends on mammalian target of rapamycin (mTOR) complex (mTORC) 1 or 2 appears to be context-dependent [54, 63, 64]. Glutamine also supports uridine diphosphate N-acetylglucosamine (UDP-GlcNAc) synthesis and subsequent N-glycosylation of lectin and mannose receptors required for pathogen recognition [25].

## Tissue macrophages and their metabolism in homeostasis

### Macrophages resident in the lung

**Fig. 2:**
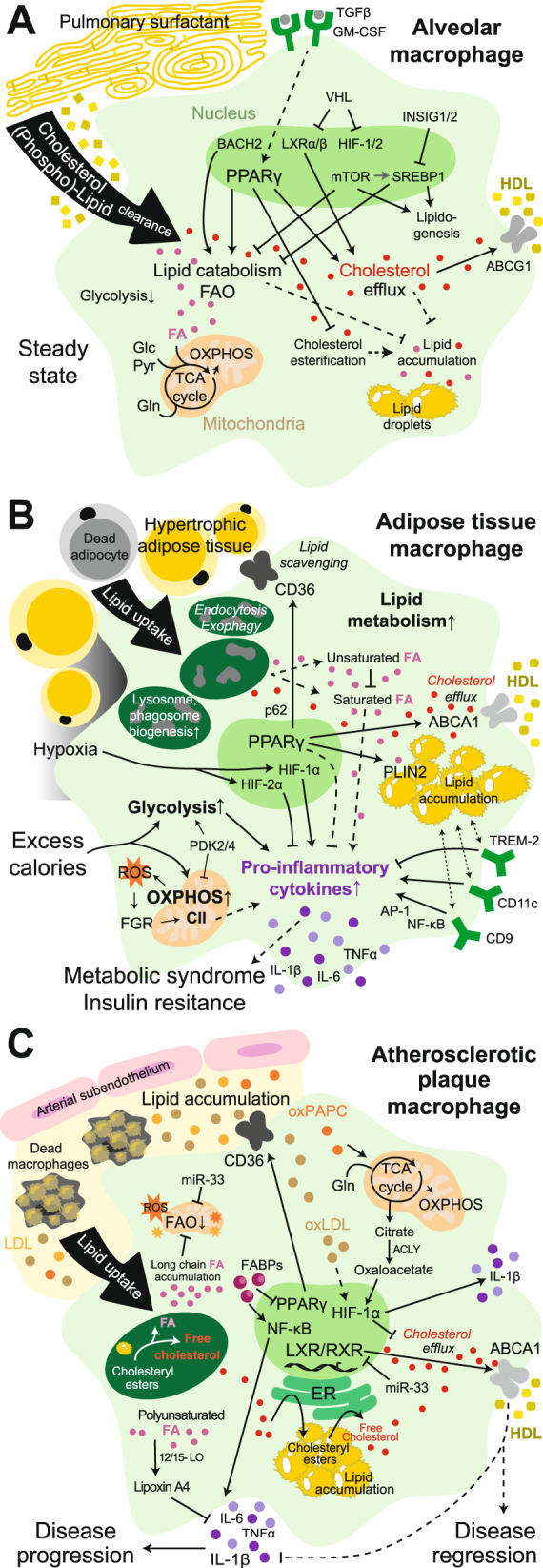
Lipid handling by tissue macrophages. **A** The metabolism of alveolar macrophages present in lung tissue is specialized for lipid catabolism and trafficking for effective clearance of pulmonary surfactant. **B** Excess calorie intake causes adipose tissue hypertrophy, hypoxia and adipocyte death. In response, adipose tissue macrophages become bioenergetically activated, scavenge resulting lipids and elevate their lipid metabolism. Ultimately, they become lipid-laden and proinflammatory and contribute to systemic metabolic syndrome and insulin resistance. **C** In atherosclerotic lesions, macrophages are exposed to a variety of lipids (i.e., oxLDL, LDL, oxPAPC, long-chain fatty acids, and cholesterol crystals) that either promote or attenuate the proatherogenic environment. Excessive free cholesterol and fatty acids, which are generated in endolysosomes upon lipid uptake, alter the metabolism of macrophages, leading to the production of proinflammatory cytokines. Conversely, effective cholesterol efflux restores macrophage functions, promoting atherosclerosis resolution. CII, complex II; FA, fatty acid; Glc, glucose; Gln, glutamine; LOX1, oxidized low-density lipoprotein receptor 1; NLRP3, NOD-, LRR- and pyrin domain-containing protein 3; Pyr, pyruvate; SRA1, steroid receptor RNA activator 1. Solid lines: direct relationships; dashed lines: indirect relationships. Purple circles: proinflammatory cytokines; gray circles: growth factors; brown and orange circles: bound cholesterol/LDL/oxLDL or oxPARC; yellow and ochre circles: bound cholesterol/HDL; red circles: free cholesterol; pink circles: fatty acids; orange stars: ROS

Alveolar macrophages (AMs, CD11c^+^ SiglecF^+^ CXC3R1^−^, Table 1) are derived from fetal liver monocytes and populate the alveolar spaces in the lung after birth, where they self-maintain during homeostasis. They phagocytose inhaled particles and perform immune surveillance, but their main physiological function is the clearance of the constantly renewed pulmonary surfactant [5, 6, 65–67]. Given that the surfactant contains ~90% lipids (mainly phospholipids and cholesterol) [68, 69], AMs are equipped for lipid catabolism and cholesterol handling [70] (Fig. 2A). Indeed, AM development from embryonic progenitors is dependent on granulocyte-macrophage (GM)-CSF- and transforming growth factor (TGF)β-mediated induction of the signature transcription factor PPARγ, a master regulator of lipid metabolism [66, 71, 72]. Alveolar type 2 epithelial cells appear to be the crucial source of GM-CSF for AM development in murine lung [73]. PPARγ-deficient AMs are dramatically reduced in number and accumulate intracellular lipids due to reduced lipid catabolism and FAO, increased cholesterol esterification and diminished cholesterol efflux. Notably, PPARγ loss alters the AM phenotype and its specific gene expression program, highlighting the fundamental importance of cellular lipid metabolism for AM identity [66, 71, 72]. Similarly, loss of BTB and CNC homology (BACH)2, a transcriptional repressor regulating immune cell development, in AMs leads to reduced expression of genes involved in lipid catabolism and transport as well as cholesterol metabolism [74, 75]. AMs also accumulate lipids in mice with enhanced activity of the transcriptional lipogenesis regulator sterol regulatory element-binding protein (SREBP)1 by loss of insulin-induced gene (INSIG)1 and INSIG2 [76]. Treatment with the SREBP1/2 inhibitor fatostatin actually decreases AM numbers in mice [77]. Consistently, SREBP1 and SREBP2 are mTOR targets, and mTOR deficiency or mTOR inhibition with rapamycin phenocopies the reductions in lung AM numbers through the regulation of lipid metabolism [77]. Likewise, deficiency of CCAAT-enhancer-binding protein (C/EBP)β, a transcription factor associated with adipocyte differentiation and lipid-induced inflammatory responses [78, 79], causes deregulated lipid metabolism and AM loss in mice [80]. Hence, a fine balance of cellular lipid metabolism appears to be vital for AM identity, function and survival [70] (Fig. 2A).

**Table 1:** Overview of the main functions and metabolic features of tissue macrophage populations

Macrophage type	Organ/system	Ontogeny	Main surface markers	Main functions	Main metabolic features	References
Alveolar macrophages	Lung	Fetal liver monocytes	CD11c^+^ SiglecF^+^ CXC3R1^−^	Surfactant clearance, phagocytosis of inhaled particles, immune sentinel functions	↑OXPHOS/mitochondrial respiration, lipid catabolism, cholesterol handling (PPARγ, LXRα, C/EBPβ, VHL); ↓Glycolysis	[5, 6, 65–70, 74–83, 85–88]
Interstitial macrophages	Lung	Adult bone marrow/blood monocytes	CXC3R1^+^ CD11b^+^ SiglecF^−^	Control of pathogens and infections, immune sentinel functions	Upon Mtb infection: ↑Glycolysis; ↓Mitochondrial respiration, fatty acid or cholesterol metabolism	[5, 65, 87, 88]
Marginal zone macrophages	Spleen	Adult bone marrow/blood monocytes	SIGNR1^+^	Removal of blood-borne antigens and pathogens	Their development and immune function is controlled by LXRα (and LXRβ)	[89–93]
Marginal metallophilic macrophages	Spleen	Adult bone marrow/blood monocytes	CD169^+^ Sialoadhesin^+^	Removal of blood-borne antigens and pathogens	Their development and immune function is controlled by LXRα (and LXRβ)	[89–93]
Tingible body macrophages	Spleen	Adult bone marrow/blood monocytes	F4/80^−^ CD68^+^	B cell phagocytosis during germinal center reaction	To be investigated	[89, 90]
Red pulp macrophages	Spleen	Yolk sac and fetal liver progenitors	F4/80^+^ VCAM1^+^ CD11b^lo^	Clearance of erythrocytes, platelets and blood pathogens; iron recycling; immune sentinel functions	↑Iron metabolism (Spi-C, NRF2, HO-1), lipid and cholesterol handling (PPARγ, LXRα)	[3, 4, 66, 70, 89, 90, 95,99–107]
Liver capsular macrophages	Liver	Adult bone marrow/blood monocytes	F4/80^+^ CX3CR1^+^ MHCII^+^	Immune surveillance, neutrophil recruitment	↓Metabolic gene signatures compared with Kupffer cells	[94, 95, 98]
Kupffer cells	Liver	Fetal liver monocytes	F4/80^+^ Clec4F^+^ Tim4^+^	Clearance of erythrocytes and blood pathogens; iron metabolism; mediators of immunological tolerance	↑Iron metabolism (Spi-C, NRF2, HO-1), lipid and cholesterol handling (PPARγ, LXRα); ↓Glycolysis (upregulated upon stimulation)	[3, 4, 7, 70, 91, 94–104, 106, 107, 109]
Erythroid island macrophages	Bone marrow	Adult bone marrow and fetal liver (likely)	F4/80^+^ VCAM1^+^ CD169^+^	Support of erythropoiesis, iron handing	↑Iron metabolism (Spi-C, HO-1) and fatty acid metabolism signatures	[106, 107]
Small peritoneal macrophages	Peritoneum	Adult bone marrow/blood monocytes	F4/80-low CD11b-low MHCII-hi	Immune sentinel functions and inflammatory regulation	↑Glycolysis and OXPHOS upon activation compared with large peritoneal macrophages	[110, 111]
Large peritoneal macrophages	Peritoneum	Yolk sac progenitors	F4/80^hi^ CD11b^hi^ MHCII^lo^	Clearance of dead cells/bacteria, inflammatory regulation, antimicrobial defense	Naïve: ↑ETC/CII, ROS, lipid and cholesterol handling (GATA6, C/EBPβ, RXRα/β) Stimulated: context-dependent OXPHOS; ↓Lipid metabolism/FAO; ↑Glycolysis	[70, 78–80, 86, 110–120]
Microglia	Central nervous system	Yolk sac progenitors	F4/80^+^ CX3CR1^+^ CD11b^+^	Immune sentinel functions; clearance of apoptotic cells; regulation of brain homeostasis, neurogenesis and synaptic activity	Naïve: ↑OXPHOS, context-dependent fuel use (mainly glucose) and metabolic pathway activation Stimulated: ↑Glycolysis; ↓OXPHOS	[121–126]
Osteoclasts	Bone marrow, spleen, blood	Adult bone marrow/blood monocytes	TRAP^+^ (tartrate- resistant acid phosphatase)	Bone resorption (dissolution of collagen and mineralized bone)	Naïve: ↑OXPHOS/CI activity, FAO, glutaminolysis Bone-exposed: ↑Glycolysis, HIF-1α, lactate production	[128–135]
Intestinal lamina propria macrophages	Intestine	Adult bone marrow/blood monocytes	CD64^+^ MHCII^hi^ CD206^+^	Clearance of dead cells, maintenance of epithelial homeostasis, immune sentinel functions, antimicrobial activity	Butyrate-exposed: ↑ROS production; Unaltered OXPHOS; ↓Glycolysis and mTOR signaling	[136–138]
Kidney-resident macrophages	Kidney	Yolk sac and/or fetal liver progenitors	CD64^+^ F4/80^+^ CD11c^+^	Clearance of dead cells, likely regulation of ureteric bud branching and vascular development	↑Fatty acid metabolism-, ↓OXPHOS- and glycolysis-related gene expression (healthy compared with lupus-like disease)	[116, 139, 140]
White adipose tissue macrophages	Lean white adipose tissue	Yolk sac progenitors (predominantly)	F4/80^+^ CD11b^+^ CD206^+^	Efferocytosis and apoptotic cell clearance	Metabolically quiescent (↓Glycolysis and ↓OXPHOS compared with macrophages from obese fat)	[189, 191, 196]
Embryonic cardiac macrophages	Heart	Yolk sac and fetal liver progenitors	CD64^+^ CX3CR1^+^	Efferocytosis and immune sentinel functions	Metabolically quiescent (↓Glycolysis and ↓OXPHOS compared with macrophages upon MI)	[141, 146]
Monocyte-derived cardiac macrophages	Heart	Adult bone marrow/blood monocytes	CCR2^+^ MerTK^+^ CD64^+^ CD11c^hi^ CD206^+^	Immune surveillance	↑Glycolysis upon MI; ↑OXPHOS and ↓Glycolysis from Day 3 after MI.	[67, 141, 146]
Skeletal muscle macrophages	Skeletal muscle	Embryonic and bone marrow precursors	CD11b^+^ F4/80^+^ CD64^+^	Maintenance of tissue homeostasis, muscle growth and regeneration	To be investigated	[145]

Interference with AM presence or AM lipid metabolism causes pathological surfactant accumulation in the lung, a rare disease termed pulmonary alveolar proteinosis [66, 71, 75], of which the inability of AMs to clear cholesterol has been suggested to be the primary underlying factor [81]. Indeed, lipid-laden macrophage-like cells are found in the alveoli of mice deficient in liver X receptor (LXR)α and β transcription factors and cholesterol/oxysterol sensors regulating reverse cholesterol transport [82] and upon loss of the LXRα target gene ATP-binding cassette (ABC) transporter ABCG1 that mediates cellular cholesterol and phospholipid efflux to high-density lipoprotein (HDL) [83]. In line with this, treatment with PPARγ agonists, LXR agonists or statins that lower systemic cholesterol levels reduces proteinosis pathology in mice [81, 84], which further supports the importance of active lipid and cholesterol metabolism in AMs (Fig. 2A).

In addition, oxygen-sensing pathways, such as von Hippel-Lindau (VHL)/HIF, regulate lipid metabolism and the identity of AMs [85]. VHL-deficient murine AMs with forced HIF-1/2-target gene expression exhibit an immature phenotype and a reduced ability to clear lung surfactant. In detail, LXR/retinoid X receptor (RXR) activation is increased in AMs upon VHL loss, resulting in increased expression of cholesterol efflux genes [85], which confirms the relevance of appropriate lipid handling for AM identity (Fig. 2A). Consistently, the homeostatic bioenergetics of AMs seem tuned toward lipid catabolism. AMs appear impaired in glycolytic metabolism, and the transcriptomic glycolysis signature is downregulated during their maturation in the lung after birth. In general, AMs display high basal respiration using glucose, glutamine, and pyruvate or fatty acid fuels, which is diminished upon VHL loss. This metabolic feature appears to be imposed upon AMs by the lung tissue microenvironment [85, 86] (Fig. 2A).

Interstitial macrophages (IntMs, CXC3R1^+^ CD11b^+^ SiglecF^–^, Table 1) are derived from blood monocytes. They are a rather scarce population mainly located between the epithelium and capillaries in the steady state, but their numbers can increase notably upon immune challenges [65]. Their predominant function is immune surveillance in the lung [5]. In agreement, in proinflammatory settings, the cellular metabolism of IntMs is reminiscent of that of M1-like BMDMs [87] (see Section 1). Analyses of *Mycobacterium tuberculosis* (Mtb)-infected murine AMs and IntMs have shown enriched OXPHOS, fatty acid metabolism and cholesterol homeostasis gene signatures as well as a higher lipid content and FAO capacity in AMs, while IntMs secrete more lactate and express higher levels of glycolysis-related genes, including iNOS [87], which inhibits OXPHOS [27, 45, 46]. These observations confirm the commitment of AMs to FAO and mitochondrial respiration, while IntMs display more glycolytic metabolism upon proinflammatory Mtb infection [87]. Nevertheless, lung macrophage populations undergo specific changes in the contexts of distinct lung diseases that also differentially affect their metabolism; this has recently been reviewed in detail [88].

### Splenic and liver macrophage populations

To date, four different macrophage types have been distinguished in the spleen in the steady state based on their locations and surface marker expression [1] (Table 1). The monocyte-derived splenic macrophage subsets comprise SIGNR1^+^ marginal zone macrophages (MZMs) and CD169^+^ sialoadhesin^+^ marginal metallophilic macrophages (MMMs) in the marginal zone as well as F4/80^−^ CD68^+^ tingible body macrophages (TBMs) in the white pulp. However, the most abundant splenic macrophage population is F4/80^+^ VCAM1^+^ CD11b^lo^ red pulp macrophages (RPMs), which are derived from the yolk sac and fetal liver progenitors and self-maintain in the splenic red pulp [89, 90]. A population of reticular fibroblasts secrete M-CSF to provide a niche for RPMs in the red pulp [9]. RPMs act as immune sentinels; however, their predominant homeostatic functions are erythrocyte and platelet phagocytosis and iron recycling [89, 90]. TBMs functionally specialize in phagocytosis of B cells that undergo apoptosis during germinal center reactions, while MZMs and MMMs harbor a predominant immunological function, capturing and clearing blood-borne pathogens such as bacteria, parasites and viruses [89]. While little is known about the metabolic features of TBMs, the development of MZMs and MMMs is controlled by LXRα/β [91]. Interestingly, rather than involving regulation of reverse cholesterol transport, the function of LXRs in macrophages of the marginal zone is connected with their immune function and clearance of phagocytosed cargo, as LXRα/β-deficient mice exhibit increased susceptibility to infection due to defective microbe control. Indeed, intracellular bacteria and apoptotic cells activate LXRs in cultured macrophages, which is required for their optimal clearance [92, 93].

In the homeostatic liver, F4/80^+^ Clec4F^+^ Tim4^+^ Kupffer cells (KCs), which form the largest tissue-resident macrophage population in the body, are found alongside F4/80^+^ CX3CR1^+^ MHCII^+^ liver capsular macrophages (LCMs, Table 1) and occasionally recruited peritoneal or other monocyte-derived macrophages. KCs originate from fetal liver monocytic precursors that colonize and self-maintain in the sinusoidal lumen, while blood monocyte-derived LCMs are present in the hepatic capsule [94, 95]. KCs can mediate immunological tolerance against blood-borne antigens from nutrients or dead cells but also clear damaged erythrocytes and circulating pathogens [95]. Upon immunogenic activation, KCs upregulate glucose uptake and pyruvate dehydrogenase kinase (PDK)-dependent glycolytic metabolism, which diminishes their tolerogenic function of IL-10 production [96, 97]. LCMs were only recently described to participate in immune surveillance and neutrophil recruitment, and apart from analysis of the enrichment of metabolic pathways in KCs compared with LCMs, their metabolism remains to be investigated [98].

**Fig. 3:**
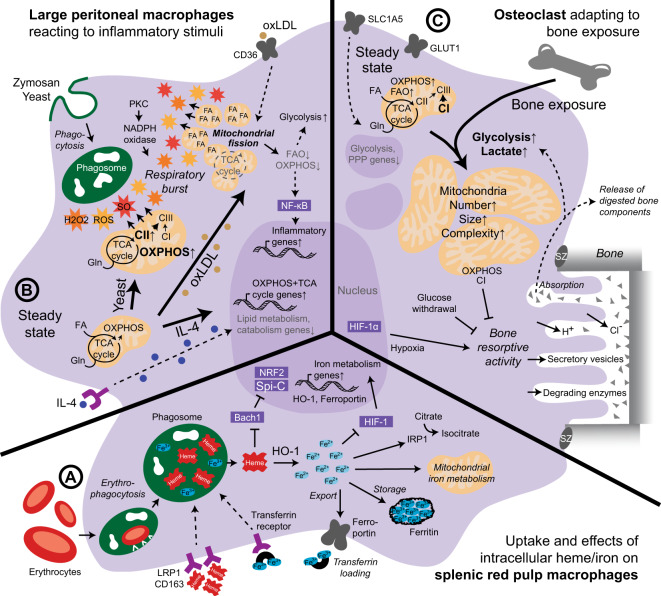
Microenvironmental influence on tissue macrophage metabolism. **A** Splenic red pulp macrophages scavenge defective erythrocytes for iron recycling. **B** Large peritoneal macrophages adapt their bioenergetics after detection of different microenvironmental factors, such as yeast, oxLDL or IL-4, to facilitate the respiratory burst. **C** Osteoclasts shift their cellular metabolism when exposed to bone, promoting bone resorptive activity. CI-III, complex I-III; FA, fatty acid; Gln, glutamine; IRP1, iron-responsive element-binding protein; PKC, protein kinase C; SO, superoxide; SZ, sealing zone. Solid lines: direct relationships; dashed lines: indirect relationships. Purple circles: cytokines; brown circles: bound cholesterol/LDL/oxLDL; red and orange stars: ROS, SO and H_2_O_2_

The bona fide macrophage populations resident in the spleen and liver, RPMs and KCs, respectively, are metabolically very dynamic cells during homeostasis and share many metabolic traits. Both populations constantly phagocytose defective erythrocytes and engage actively in the recycling, storage and metabolism of iron [89, 95, 99]. Recently, iron metabolism in macrophages was the subject of several reviews and is summarized here [100–103]. Molecularly, most genes involved in iron handling are induced by Spi-C and nuclear factor erythroid 2–related factor (NRF)2 transcription factors, both of which are expressed by murine RPMs and KCs [99, 104]. During in vivo differentiation of new resident macrophage populations from monocytes after their loss, Spi-C expression is induced in RPMs by the metabolite heme [105]. In differentiating KCs, Spi-C is mediated by Notch ligands derived from liver sinusoidal endothelial cells within a KC niche that also comprises stellate cells and hepatocytes [7, 8]. Spi-C and NRF2 are, however, repressed by BACH1 (Fig. 3A). Intracellular iron or heme, which is recycled from erythrocytes or taken up by RPMs and KCs via various receptors, such as the hemoglobin scavenger receptor CD163 or LDLR-related protein 1 (LRP1), sequesters BACH1, resulting in the expression of genes required for iron metabolism, including heme oxygenase 1 (HO-1), ferroportin and ferritin. HO-1 degrades heme using oxygen and NADPH to release iron into the cytoplasm, where it is stored by ferritin or regulates cellular metabolic processes such as HIF signaling, steps of the TCA cycle via iron-responsive element-binding protein 1 or mitochondrial iron metabolism. Iron is exported from macrophages by ferroportin located in the plasma membrane and, once extracellular, loaded onto transferrin [100–103] (Fig. 3A). In the bone marrow, erythroblasts take up transferrin-bound iron, which is aided by F4/80^+^ VCAM1^+^ CD169^+^ erythroid island macrophages (EIMs) that specialize in iron handling [106]. Indeed, HO-1-deficient mice display a notable loss of macrophages in the spleen, liver and bone marrow [107]. Spi-C has long been known to be fundamental for RPM and EIM development, and Spi-C loss reduces the numbers of these cells but, interestingly, does not affect KCs. The remaining Spi-C-deficient RPMs exhibit an impaired ability to phagocytose and clear erythrocytes [104, 105]. KCs, however, also regulate systemic iron metabolism via their ability to suppress hepatocyte-expressed hepcidin, which causes degradation of ferroportin upon high plasma iron levels and limits iron export from macrophages [99, 102].

In addition, evidence points toward active lipid and cholesterol handling of murine hepatic KCs and splenic RPMs, primarily their strong lipid metabolism signature compared with that of other macrophage populations. In detail, both cell types express notable levels of PPARγ and LXRα as well as many of their target genes involved in lipid metabolism and cholesterol trafficking [3, 4, 70, 99, 108]. SREBP1 and SREBP2, which are LXR target genes that control fatty acid and cholesterol synthesis, are also expressed by KCs [109]. Similar to Spi-C expression, LXRα expression is also induced in differentiating KCs by liver sinusoidal endothelial cells of the KC niche via Notch ligands. Notably, LXRα appears to be the transcription factor with the highest expression in KCs and has been described to be KC lineage determining [7, 8]. Indeed, whereas LXRα/β deficiency does not affect RPM presence [91], a lack of LXRα results in a mixed population of embryonic KCs and monocyte-derived cells, stressing the importance of LXRα for KC identity [7, 108]. On the other hand, KCs appear unaltered by PPARγ deletion, while the differentiation of RPMs is notably compromised upon PPARγ loss [66]. Nevertheless, apart from comparison of the transcriptomic signatures, the actual metabolic activity of KCs or RPMs handling lipids and cholesterol or its relevance for the functions of these cells in homeostasis remains largely unclear. Intriguingly, there may be a connection between iron and lipid/cholesterol metabolism in KCs and RPMs, as the transcriptome of bone marrow EIMs, which shows their specialization in iron handling, also reveals a strong fatty acid metabolism signature [106].

### Peritoneal macrophages

During homeostasis, exudates of the peritoneum contain two main macrophage populations, F4/80^hi^ CD11b^hi^ MHCII^lo^ large peritoneal macrophages (LPMs) and F4/80^lo^ CD11b^lo^ MHCII^hi^ small peritoneal macrophages (SPMs, Table 1). LPMs originate from yolk sac progenitors, self-maintain, and are involved in dead cell/bacteria phagocytosis and inflammatory responses. SPMs, which are constantly differentiated from blood monocytes, act as potent immune sentinels and inflammatory regulators [110]. LPMs are more numerous than SPMs in the steady state; however, the presence of SPMs increases profoundly upon stimulation or inflammation, when SPMs out-populate LPMs in a process termed the “macrophage disappearance reaction” [110]. In a comparative study, thioglycolate-elicited SPMs exhibited not only an elevated activation status and activity but also a much higher extracellular acidification rate (ECAR) and OCR than resident LPMs [111]. This fact has to be taken into consideration when examining features of different peritoneal macrophage populations, including their metabolism.

The metabolic state of murine LPMs supports the induction of immune responses and antimicrobial defense. This state involves elevated mitochondrial and ETC activity linked to mtROS production upon inflammatory stimulation, pointing toward a role of LPMs in controlling infection [112, 113] (Fig. 3B). LPMs display a high oxidative respiration rate fueled by glutamine and fatty acids in the steady state compared with that of BMDMs or AMs [86, 114, 115] but not with that of elicited SPMs [111]. In fact, the high levels of the metabolite glutamate in the peritoneum have been proposed to be a “tissue-niche fuel” for LPMs [114]. Upon stimulation of LPMs with zymosan or yeast, mitochondria are recruited to phagosomes contributing to an antimicrobial respiratory burst, specifically via glutaminolysis-mediated induction of ETC complex II [114] (Fig. 3B). LPMs also appear very responsive to in vivo stimulation with the canonical type 2 cytokine IL-4 and, notably, further upregulate genes involved in OXPHOS and the TCA cycle, while genes involved in lipid metabolism, such as those in the PPARγ signature, are downregulated [86] (Fig. 3B).

In contrast, exposure of thioglycolate-elicited murine SPMs and LPMs to a distinct class of stimulants such as CD36-binding oxidized low-density lipoprotein (oxLDL) results in reductions in FAO and OXPHOS and upregulation of glycolysis in concert with increased nuclear factor kappa-light-chain-enhancer of activated B cells (NF-κB) activation and inflammatory cytokine production [115]. Stimulation of LPMs with ovalbumin opsonized with IgG has a similar effect decreasing the OCR [116]. However, oxLDL-induced inflammatory activation is in fact dependent on a primary repurposing of the ETC toward ROS and superoxide production, with oxLDL promoting structural changes in the mitochondria which suppress OXPHOS. The timing suggests the oxLDL-mediated glycolytic switch in peritoneal macrophages to be a secondary effect following the metabolic adaption of mitochondrial structure and functionality to facilitate ROS generation [115] (Fig. 3B).

In addition, studies on the LPM-expressed signature transcription factors GATA6, C/EBPβ and RXRα/β suggest a relevance of lipid handling for LPM identity that is in line with the functions of LPMs as phagocytes [80, 117–119]. LPMs express higher levels of proteins involved in lipid handling and cholesterol transport than RPMs [120]. GATA6-deficient LPMs display an even further increased OCR, in line with their observed alternative activation, and diminished aspartoacylase expression. Aspartoacylase can regulate acetyl-CoA formation as well as lipid synthesis, and aspartoacylase-mutant LPMs are more prone to death than their wild-type counterparts [117]. In accordance, C/EBPβ controls lipid metabolism-related genes in LPMs, and C/EBPβ-deficient LPMs exhibit striking upregulation of LXRα and PPARγ [70, 78, 79]. Moreover, loss of RXRs in LPMs also results in an enhanced lipid metabolism signature and lipid accumulation, especially via lipid-containing lysosome-like vesicles [119]. Notably, all three transcription factors have been reported to be vital for murine LPM differentiation, survival, maturation and polarization [80, 117–119]. In line with this, mixed thioglycolate-elicited SPMs and LPMs show a strong TCA cycle, FAO and FAS transcriptional profile [115].

### Microglia in the central nervous system

F4/80^+^ CX3CR1^+^ CD11b^+^ microglia originate from embryonic yolk sac macrophages and reside in the central nervous system, where they self-maintain (Table 1). They function as immune sentinels to protect the brain from pathogens but are also required to maintain brain homeostasis by regulating neurogenesis, synaptic activity and apoptotic cell clearance [121]. Their intrinsic metabolism has been reviewed recently [122–124] and is summarized here.

Microglia engage in active mitochondrial OXPHOS metabolism to meet their energetic demands in the steady state and undergo a glycolytic switch upon activation, similar to cultured BMDMs. In the mouse brain, compared to astrocytes and neurons, microglia express similar levels of TCA cycle and glycolytic genes but higher levels of OXPHOS-related genes. Microglia also express factors involved in glucose uptake, which appears to be required for ROS production [122–124]. Ex vivo, untreated and anti-inflammatory (IL-13+IL-4)-stimulated microglia show high basal and maximal respiratory rates, while proinflammatory (LPS + IFNγ) stimulation collapses the OCR of microglia and reduces the OCR/ECAR ratio, suggesting a glycolytic switch [125].

Murine microglia have striking metabolic plasticity, adapting their OXPHOS activity to their microenvironment, for example, the presence of serum in culture medium [125]. Furthermore, using in vivo and in situ imaging of intracellular NADPH, microglia have been shown to adjust to hypoglycemia/aglycemia and switch from glucose as their main fuel to glutamine to support their OXPHOS metabolism, a process requiring mTOR [126]. As microglia are in constant communication with their microenvironment, numerous metabolic pathways are involved in their function, such as uptake and metabolism of free fatty acids/FAO, lactate and ketone bodies [122–124, 126].

Finally, an energy-deficient state of microglia in the aging mouse brain fosters inflammation. During aging, the prostaglandin E2/EP2 signaling axis causes reduced glycolytic flux and OXPHOS in microglia via glucose sequestration into glycogen. Blocking the EP2 cascade rescues bioenergetics in microglia, ameliorates aging-associated neural inflammation and improves memory [127]. These findings highlight the importance of functional energy metabolism in a tissue macrophage population for the maintenance of neural homeostasis.

### Osteoclasts in mineralized bone

Osteoclasts, which are present in the bone marrow, spleen and blood, are multinucleated and terminally differentiated from monocytes (Table 1). They specialize in bone resorption and dissolve collagen and mineral bone matrix by releasing proteolytic enzymes and acids. As this activity comes with high bioenergetic demands, mature human and mouse osteoclasts contain more mitochondria than other cell types, and these mitochondria are of greater size and complexity [128] (Fig. 3C).

Murine osteoclastogenesis is driven by osteoblasts producing receptor activator of nuclear factor kappa-Β ligand (RANKL) and osteoprotegerin (OPG), which is termed the RANK-RANKL-OPG system [6]. In vivo and in vitro, this system seems to rely on mitochondrial biogenesis and OXPHOS, especially complex I of the ETC, which is regulated by iron uptake, PGC-1β and alternative NF-κB (RelB and NIK) signaling [129–131]. In addition, human and mouse osteoclast differentiation with RANKL and M-CSF induces an ECAR decrease and increases the gene expression of ETC components and the mitochondrial OCR [131–133]. In line with this, hypoxic conditions limit human and murine osteoclastogenesis in vitro [128], and in human bone sections, osteoclasts are associated with high FAO activity and low expression of glycolytic or PPP enzymes [134]. OXPHOS is likely fueled by glutaminolysis (Fig. 3C) and controlled by c-Myc. This is evidenced by the upregulation of glutamine importer solute carrier family 1 member 5 (Slc1a5) and glutaminase-1 during osteoclastogenesis and the fact that glutamine withdrawal as well as inhibition of Slc1a5 or c-Myc reduces osteoclast differentiation [135]. Upregulation of glycolytic genes/glucose transporters and suppression of osteoclast differentiation by mTOR inhibition or 5ʹ AMP-activated protein kinase (AMPK) activation have also been observed [135]. However, osteoclastogenesis has been reported to be unaffected by 2-deoxy-d-glucose [132] and reduced by lactate [129]. This discrepancy is likely explained by the metabolic switch of osteoclasts toward glycolysis upon bone absorption [128] (Fig. 3C). Activation of murine osteoclasts with bone powder does induce enhanced glycolytic activity compared to that in unstimulated cells, and the bone resorption activity of osteoclasts is driven by enhanced glycolysis, HIF-1α and lactate production [132, 135]. In addition, the collagen degradation activity of human osteoclasts is diminished when the cells are cultured in the absence of glucose, which enforces an OXPHOS-driven metabolism. In turn, complex I inhibition by rotenone augments the resorptive function of osteoclasts [133].

### Other macrophage populations

Intestinal macrophages residing in the lamina propria are exposed to numerous nutrients and metabolites during homeostasis that influence their metabolism and activities, primarily microbiota-derived factors [136]. The functions of monocyte-derived CD64^+^ MHCII^hi^ CD206^+^ lamina propria macrophages include apoptotic cell removal, promotion of epithelial integrity, immunoregulation and antimicrobial activity [137]. For example, microbiota-derived butyrate triggers enhanced ROS production in murine macrophages, while OCR is unaltered and glycolysis and mTOR signaling are inhibited, resulting in increased bactericidal functions [138] (Table 1).

The predominant macrophage type in the kidneys appears to be embryo-derived CD64^+^ F4/80^+^ CD11c^+^ kidney resident macrophages (KRMs). Functionally, they contribute to dead cell clearance, ureteric bud branching and likely vascular development [139, 140]. Murine KRMs appear metabolically quiescent in the steady state. In comparison, KRMs from mice with lupus erythematosis-like disease display stronger OXPHOS and glycolysis-related, but weaker fatty acid metabolism, gene signatures compared with disease-free mice (Table 1). Inhibition of this glycolytic switch by KRMs upon disease may represent a therapeutic approach controlling kidney inflammation [116]. Interestingly, human synovial macrophages from patients with rheumatoid arthritis, which is also an immune complex-associated disease, show higher glycolytic gene expression but weaker OXPHOS and fatty acid metabolism signatures than those from healthy donors [116].

Overall, various populations of macrophages (co-)exist throughout the body and specialize in distinct, mostly tissue-specific activities. While these populations undoubtedly have to adapt to changing environments, the diverse metabolic programs of macrophages in tissues emerge to be perfectly optimized to facilitate the homeostatic functions of these cells and contribute to their identities (Table 1).

## Macrophage metabolism in tissue regeneration

Upon tissue damage, macrophages play an active role in the early initiation of acute inflammation and later in the anti-inflammatory phase of cell proliferation and remodeling associated with the resolution of inflammation (Fig. 1B). After the initial injury, embryo-derived tissue-resident macrophages are rapidly replaced by monocytes [141, 142], which show a switch toward an alternatively activated anti-inflammatory state within the first days after damage. These monocyte-derived macrophages infiltrating tissues upon sterile injury drive tissue regeneration, which is impaired in mice lacking CCR2 [143]. Upon resolution of inflammation, steady-state self-maintenance of macrophages is recovered [144].

Most studies addressing the importance of macrophage metabolism in tissue regeneration use skeletal muscle injury or myocardial infarction (MI) models. CD11b^+^ F4/80^+^ CD64^+^ macrophages are present in skeletal muscle during homeostasis and stem from embryonic and adult bone marrow precursors, showing remarkable diversity, with at least 4 different subsets identified using single-cell RNA sequencing [145]. CD64^+^ CX3CR1^+^ resident cardiac macrophages originate from the yolk sac and fetal liver progenitors, but monocyte-derived macrophages are also present in the heart (Table 1). Functionally, they clear dead cells and act as immune sentinels with pro- and anti-inflammatory functions in the steady state; however, little is known about their cellular metabolism and how it affects their functions in homeostasis and upon injury [146].

### Metabolic changes in macrophages after tissue injury

Damage-associated molecular patterns released from dead cells upon sterile tissue injury activate macrophages via pattern recognition receptors. This activation is linked to a rewiring of cellular metabolism and happens along with changes in nutrient availability that may both precede and be the consequence of the metabolic changes taking place. An integral view of how metabolism regulates injury and repair has been provided elsewhere [147]; here, we will focus on the intrinsic changes in macrophage metabolism during tissue regeneration.

Within 24 h upon proinflammatory activation, macrophages enhance their glycolytic metabolism [21] (Fig. 1B). This change has been confirmed in an indirect manner via transcriptomic analysis in models of MI or skeletal muscle repair, in which upregulated glycolysis and hypoxia response signatures have been found in macrophages one day after tissue injury [141, 142]. The hypoxic environment caused by skeletal muscle injury is maintained for several days with a peak on Day 4 [148]. The infiltration of proinflammatory macrophages in the tissue is HIF-dependent, as shown in mice with specific deletion of HIF-1α and HIF-2α in lysozyme M (LysM)-expressing cells [148, 149]. Although HIF is reported to be an essential transcription factor for M2 polarization upon hypoxia/lactic acid/IL-4 sensing [150], the deletion of HIF does not affect the shift of macrophages toward an anti-inflammatory phenotype in the context of skeletal muscle regeneration, as indicated by typical M2 marker expression in vitro and in vivo [148]. In line with this, tissue regeneration was not affected by HIF deletion in LysM-expressing cells in two models of muscle injury [148] and was only slightly affected in a third model of mild tissue trauma [149]. These observations imply that although the macrophage transition from a proinflammatory to a resolutive state takes place in a hypoxic environment, it is independent of HIFs. This finding correlates with the rapid decline in HIF expression by macrophages 3 days after MI [141]. Consistent with HIF expression, following an initial glycolytic burst on Day 1, glycolytic genes are promptly downregulated at Days 2-3 (Fig. 1B), as shown by two studies that longitudinally analyzed the transcriptomes of macrophages after MI [141] and muscle injury [142].

### Switch of macrophage metabolism for tissue repair and remodeling

As early as 24–72 h upon tissue injury, macrophage function changes toward an anti-inflammatory phenotype that promotes cell proliferation and tissue remodeling. At this time, glycolysis-related genes are promptly downregulated [141, 142], while mitochondrial metabolism-related genes (including TCA cycle and ETC genes) are upregulated, an effect that is sustained during the tissue regeneration phase [142]. In line with the in vitro findings, additional evidence supports the role of mitochondrial metabolism in the anti-inflammatory function of macrophages during tissue repair (Fig. 1B).

First, AMPK1α, a key metabolic enzyme that can enhance OXPHOS, increases its activity in macrophages shortly upon tissue injury and is essential for the anti-inflammatory phenotype of macrophages and appropriate muscle regeneration [151]. Furthermore, a key function of macrophages in terminating the production of inflammatory mediators and promoting inflammation resolution is efferocytosis, which is required for the production of anti-inflammatory cytokines [152]. A growing body of literature shows that this process depends on metabolic rewiring, which, after an early phase that is dependent on increased glucose uptake and aerobic glycolysis [153], focuses on mitochondrial and fatty acid metabolism (Fig. 1B). In an in vivo model of apoptotic cell clearance in the thymus upon dexamethasone treatment, mitochondrial uncoupling protein 2 [154] and dynamin-related protein 1 [155], which reduce the mitochondrial membrane potential and mitochondrial fission, respectively, were found to be required for effective and continuous efferocytosis by macrophages. The catabolism of phagocytosed apoptotic cells by macrophages leads to an increase in the OCR fueled by FAO. In this context, ETC dysfunction by depletion of Rieske iron-sulfur protein (RISP), an essential subunit of mitochondrial complex III, in LysM-expressing cells in mice leads to a decrease in IL-10 expression that translates into a loss of ventricular systolic function upon MI [156]. IL-10 expression upon ETC interference in myeloid cells can be rescued with the NAD^+^ precursor nicotinamide mononucleotide [156], which has been found to be cardioprotective in an ischemia and reperfusion model [157]. For instance, hydrogels loaded with glutamine may increase mitochondrial spare respiratory capacity, and the administration of nicotinamide mononucleotide preclinically improves recovery upon sterile tissue injury [156–158]. In addition, IL-10 production is increased in macrophages from nonhypoxic regions of the heart, suggesting that the progressive recovery of oxygen supply in the tissue may work as an environmental cue that promotes tissue regeneration [156].

The significant external lipid substrate provided by apoptotic cells also increases the expression of PPARδ in macrophages, which increases opsonin expression and activates a transcriptional program required for apoptotic cell clearance and anti-inflammatory gene expression [159]. Ly6C^−^ macrophages, which are anti-inflammatory, also show high expression of PPARγ upon tissue injury [160] (Fig. 1B). While alternative macrophage polarization in vitro is regulated by PPARγ [57], macrophages lacking PPARγ can transition toward an anti-inflammatory phenotype in vivo, as Ly6C^+^ and Ly6C^−^ macrophages have been found to appear over time in a model of skeletal muscle damage [160]. However, PPARγ mediates the transcriptional control of growth factors produced by macrophages that can regulate skeletal muscle regeneration, which is subsequently impaired in mouse models with PPARγ-deficient macrophages [160]. Consistent with this, longitudinal mass spectrometry-based lipidomics during the transition from inflammation to resolution and regeneration in skeletal muscle injury have indicated that macrophages are both sources and sensors of lipid mediators. These factors play a role in the temporal transition from Ly6C^hi^ pro-inflammatory to Ly6C^lo^ pro-resolution macrophages. In particular, macrophage production of polyunsaturated fatty acid derivatives such as resolvins, which are specialized pro-resolving mediators, increases over time during tissue repair [161] (Fig. 1B). Resolvin D2 is first expressed in Ly6C^lo^ macrophages at Day 2 upon tissue injury; at the same time, its receptor G protein-coupled receptor 18 is highly expressed on Ly6C^hi^ macrophages, suggesting directional anti-inflammatory signaling cues. In line with this, the expression of lipoxygenases, the key enzymes for polyunsaturated fatty acid metabolism, is also increased in macrophages in the resolution phase [161].

Regarding amino acid metabolism, transcriptomic analyses have shown increases in glutamine metabolism genes in macrophages at the late stages of tissue recovery [142]. Muscle injuries are characterized by low glutamine levels; however, macrophages synthesize and secrete glutamine to promote the growth of satellite cells and improve muscle regeneration. Furthermore, macrophage-targeted inhibition of glutamine oxidation by glutamine dehydrogenase-1 improves muscle regeneration in muscle injury and ischemia models [162]. In addition, while proinflammatory macrophages use arginine for the generation of NO via iNOS [163], a diverging pathway competes for arginine during tissue remodeling. Arginase-1 catalyzes the production of ornithine by BMDMs, which is used as a substrate for the synthesis of the collagen precursor proline and polyamines, which support mitochondrial metabolism in alternatively activated macrophage models [52]. Hence, arginase-1 activity in macrophages may provide the extracellular matrix and promote the proliferation of stromal and satellite cells during wound healing [164, 165] (Fig. 1B).

### Metabolic defects in macrophages in unresolved tissue regeneration

Macrophages play key roles in models of unresolved tissue injury, such as idiopathic pulmonary fibrosis [166]. AMs from fibrosis murine models first increase glycolysis and then switch metabolically to FAO, as tested by extracellular flux analysis and as indicated by the increased expression of key enzymes [167, 168]. The FAO increase is dependent on the mitochondrial calcium uniporter (MCU) and mtROS increase-driven expression of PGC-1α. Indeed, blockade of this mechanism in AMs causes metabolic reversal to glycolysis and protects mice from fibrosis [168]. Itaconate and its synthesizing enzyme immune-responsive gene 1 (IRG1) are reduced in AMs from idiopathic pulmonary fibrosis patients. Consistently, AMs lacking itaconate are more profibrotic than those with itaconate and lead to exaggerated persistent fibrosis. In addition, itaconate administration is preclinically used for the treatment of fibrosis, providing further evidence of its immunoregulatory role [169].

Iron concentrations are increased in bronchoalveolar lavage fluid and AMs from idiopathic pulmonary fibrosis patients, and these increases are associated with a proinflammatory phenotype and ROS production [170, 171]. In addition, an increased frequency of transferrin receptor 1 (CD71)^−^ macrophages, which are characterized by the expression of profibrotic genes, is inversely correlated with survival in these patients [172]. Strategies to improve lung fibrosis using drugs that affect metabolism have been tested in the clinic. However, reduction of ROS via N-acetylcysteine (clinical trial identifier NCT 00650091) or the use of metformin [173], which was efficient in preclinical research due to AMPK activation [174], fails to impact clinical outcomes. Notably, none of these strategies are specifically directed toward macrophages or based on macrophage-related immune-metabolic evidence.

Altogether, these data suggest that the targeted manipulation of cellular metabolism in macrophages is a promising target to increase the speed of wound healing and prevent inadequate tissue regeneration like that in fibrosis [88].

## Metabolic adaptations of adipose tissue macrophages upon overnutrition

Nutritional challenges, such as refeeding after starvation or excess calorie intake, systemically affect the activities and metabolism of macrophages in tissues, including white adipose tissue (WAT), pancreas, liver, peritoneum and brain. Prolonged diet-driven perturbations of tissue macrophages are associated with pathologies such as type II diabetes, nonalcoholic fatty liver disease (NAFLD) and cancer [99, 175–179]. During obesity, persistent overnutrition causes lipid accumulation and hypertrophy of WAT. The imposed mechanical stress, as well as oxygen shortage, results in adipocyte death. In conjunction, this activates adipose tissue macrophages (ATMs) to secrete proinflammatory mediators such as tumor necrosis factor (TNF)α or IL-1β that, in turn, stimulate inflammatory pathways such as the c-Jun N-terminal kinase (JNK) or inhibitor of nuclear factor kappa-B kinase subunit (IKK)β pathways in adipocytes. These mechanisms interfere with insulin signaling, culminating in insulin resistance, lipid accumulation in the liver or NAFLD and metabolic syndrome [175–177]. Liver-resident macrophages, such as KCs or monocyte-derived macrophages, react to systemic inflammation, gut-derived metabolites and other factors during NAFLD and can foster its progression to nonalcoholic steatohepatitis (NASH), fibrosis and liver cirrhosis. In livers with NASH, causative remodeling of macrophage subpopulations and a shift toward a proinflammatory phenotype have been reported, which makes macrophages a promising target in NAFLD/NASH. This phenomenon and the underlying metabolic alterations in liver macrophages have been the subjects of several recent reviews [70, 95, 99, 109, 175, 180–182]. Hence, here, we focus on the interplay of metabolic and functional adaptations of ATMs upon overnutrition that promote obesity and metabolic syndrome (Fig. 2B).

Overall, in lean or obese WAT, there are distinct ATM populations associated with proinflammatory functions (lipid-laden CD9^+^ ATMs, CD11c^+^ ATMs), anti-inflammatory functions (TREM-2^+^ lipid-associated macrophages [LAMs], vasculature-associated macrophages, Txnrd1^+^ HO1^+^ Mox-like macrophages) or dual functions (glucose+insulin+palmitate-treated BMDMs [MMe-like ATMs]) [183–189]. Moreover, sympathetic neuron-associated macrophages that accumulate in hypertrophic WAT and produce TNFα and IL-1α [190] as well as homeostatic Ly6C^+^ ATMs with an anti-inflammatory profile [183] have been described.

### Bioenergetic activation of adipose tissue macrophages upon excess calorie intake

The key role of ATMs in obesity-associated low-grade inflammation has long been known to be driven by notable changes in the cellular metabolism of ATMs. ATMs from obese WAT display a glycolytic and proinflammatory state. However, comparisons of lean vs. obese ATMs and unstimulated vs. LPS-activated BMDMs have revealed that ATMs have different transcriptomic and proteomic profiles than M1-polarized macrophages, both in mice and human [183, 187, 191, 192]. This highlights the uniqueness and complexity of the metabolic and functional states of distinct ATM populations upon overnutrition.

Healthy WAT predominantly contains F4/80^+^ CD11b^+^ CD206^+^ anti-inflammatory or redox-regulatory (Txnrd1^+^ HO1^+^ Mox) macrophages derived from the embryonic yolk sac, with some contribution of monocyte precursors [193, 194] (Table 1). In addition, homeostatic WAT contains embryo-derived vasculature-associated ATMs, which associate with blood vessels, endocytose blood-borne macromolecules and are filled with lipid droplets [186]. Lean ATMs have been proposed to engage in OXPHOS/FAO-driven metabolism [195]. Yet, ATMs in lean mice appear metabolically quiescent with low OXPHOS and glycolytic gene expression and a low OCR and ECAR. Rapid bioenergetic/metabolic activation of murine ATMs is observed upon high-fat diet (HFD) feeding, as evidenced by increases in OCR, ECAR and lactate release as well as glycolysis and OXPHOS gene expression signatures [189, 191, 196]. CD14^+^ myeloid cells from visceral WAT of obese patients with diabetes display a comparable metabolic activation, in contrast to myeloid cells from nondiabetic obese individuals [191]. Moreover, co-culture of BMDMs with lean or obese WAT has corroborated the bioenergetic metabolic activation of ATMs upon HFD feeding [191]. WAT expression of FGR kinase (a tyrosine kinase from the Src family), mainly by macrophages, is also linked to obesity, liver steatosis and insulin resistance [197]. MtROS generated in stressed ATMs activate FGR, which mediates mitochondrial complex II activation and is associated with proinflammatory cytokine production [37, 41, 197] (Fig. 2B). Thus, FGR deletion in bone marrow-derived cells prevents insulin resistance and liver steatosis upon HFD feeding in mice [197]. In one study, obesity-associated pathologies were also ameliorated in mice treated with the near-infrared fluorophore IR-61, which preferentially targets macrophages, increasing OXPHOS and ameliorating WAT inflammation [198]. However, the effect of IR-61 on mitochondrial metabolism of adipocytes, whose impairment is a hallmark of obesity in mice [199], was unfortunately not investigated in that study.

The unique metabolic activation of ATMs during diet-induced obesity can largely be ascribed to characteristics of the dramatically expanded WAT microenvironment, the creation of hypoxia due to inadequate angiogenesis, the release of danger signals by dying adipocytes and the abundance of (adipocyte-derived) lipids and fatty acids [175, 176, 193]. However, changes in the bioenergetics of ATMs may also be influenced by the almost exclusive monocytic origin of ATMs in obese mice [189, 193, 194].

### Hypoxia, HIF, glycolysis and the PPP in adipose tissue macrophages

Upon refeeding after fasting, murine ATMs in lean WAT, in contrast to several other types of tissue macrophages, induce a proinflammatory IL-1 pathway response and a transcriptional lipid metabolism signature (PPAR signaling, glycerolipid metabolism, fatty acid degradation) [178]. Inhibition of glycolysis, glutaminolysis and FAO reduces proinflammatory cytokine secretion by cultured lean ATMs, while only glycolysis drives the enhanced release of IL-6 and CXCL1 by ATMs from obese WAT [191]. Hypoxia sensing and proinflammatory stimulation associate with the glycolytic metabolism of ATMs during overnutrition (Fig. 2B). Murine ATMs in obese compared with lean WAT display enhanced activation of proinflammatory HIF-1α, resulting in the expression of IL-1β and glycolytic genes [196]. Consistent with HIF-1α driving a glycolytic metabolism and proinflammatory state, ATM accumulation and transcription of IL-1β appear to be reduced in WAT of HFD-fed LysM-Cre HIF-1α^f/f^ mice which also exhibit mildly improved glucose tolerance and increased expression of angiogenic factors. However, the effects of HIF-1α loss in ATMs on general adipose tissue inflammation, angiogenesis, adiposity or insulin sensitivity are context-dependent [191, 196, 200]. Supporting the dependence of proinflammatory functions of ATMs on their glycolytic metabolism, HFD-fed mice with PDK2/4-deficient bone marrow display lower ATM numbers and inflammation (TNFα, IL-6 and CCL2 expression) in WAT as well as ameliorated insulin resistance [201].

In contrast, HIF-2α expression, also observed in lean and obese ATMs, is rather associated with an inflammation-resolving (M2-like) ATM state, reduced expression of TNFα or IL-12 [202] and ameliorated inflammasome activation. In BMDMs and peritoneal macrophages, HIF-2α deficiency enhances the OCR and FAO via carnitine palmitoyltransferase 1 A upregulation during inflammasome activation, which drives IL-1β and IL-18 secretion [203]. Adipocyte and peritoneal macrophage co-cultures have confirmed the anti-inflammatory role of HIF-2α in macrophages, which includes the induction of arginase-1 and limitation of NO and proinflammatory gene expression in adipocytes [202]. HIF-2α^+/–^ mice indeed display ATM accumulation, insulin resistance and susceptibility to adipose tissue inflammation (TNFα and IL-6) upon overnutrition [202]. HFD-fed LysM-Cre HIF-2α^f/f^ mice also exhibit signs of metabolic syndrome and elevated IL-1β and IL-18 levels in plasma. Notably, stabilization of HIF-2α by treatment with its agonist FG-4592 alleviates overnutrition-induced inflammasome activation and insulin resistance [203].

Finally, activation of the PPP is also associated with proinflammatory features of macrophages during excess calorie intake. First, glucose-6-phosphate dehydrogenase (G6PD), the initial enzyme of the oxidative branch of the PPP, is highly expressed in ATMs from obese compared with lean mice, and its levels in human adipose tissue correlate with several parameters of obesity [204]. G6PD-deficient ATMs from obese mice exhibit decreased TNFα and CCL2 expression, and G6PD-deficient peritoneal macrophages are less responsive to LPS stimulation ex vivo than their wild-type counterparts. Indeed, glucose intolerance; crown-like structure (CLS) number; and TNFα, IL-6 and CCL2 levels in adipose tissue are improved in HFD-fed mice grafted with G6PD-deficient bone marrow compared with controls [205]. Second, the potential involvement of sedoheptulokinase, which is part of the nonoxidative branch and negatively regulates the flux through the PPP by producing sedoheptulose 7-phosphate, in overnutrition-induced pathologies has also been proposed. Sedoheptulokinase is expressed by M2-like macrophages in vitro and downregulated to allow proinflammatory M1-like macrophage features [206].

### Lipid handling by adipose tissue macrophages

The metabolic state of ATMs in expanded WAT is affected by dietary or adipocyte-derived fatty acids [207]. ATMs accumulate in CLS around dying adipocytes in obese WAT and clear released lipids via endocytosis or larger particles via exophagy, causing foam cell formation [208]. Enhanced lipid uptake and lipid droplet formation are linked to lysosomal biogenesis in murine ATMs from obese WAT, and ATMs accumulate more lipids upon inhibition of lysosome function [192]. Herein, we outline the key concepts of lipid metabolism in ATMs investigated in vivo (Fig. 2B); while adaptations of macrophages after fatty acid/lipid exposure have been recently reviewed [207, 209].

Full-length oxidized phospholipids, which are predominantly found in WAT of HFD-fed mice, augment BMDM bioenergetics and proinflammatory gene expression [189]. In contrast, truncated oxidized phospholipids diminish the OCR and ECAR of cultured BMDMs while increasing the expression of Mox-like antioxidant genes. Moreover, (long-chain) saturated fatty acids stimulate not only glycolysis, lipid metabolism and OXPHOS in macrophages but also a switch to a proinflammatory phenotype [209]. Indeed, murine BMDMs increase glycolysis and HIF-1α expression as well as the basal OCR when exposed to the saturated fatty acid palmitate, which culminates in IL-1β induction [196]. Conversely, unsaturated fatty acids show anti-inflammatory effects, even opposing saturated fatty acids [209]. TLR4-dependent priming of BMDMs that causes alterations in cellular metabolism, lipid handling and membrane composition is required for the proinflammatory effects of palmitate [210].

However, palmitate treatment of BMDMs does not entirely reproduce the bioenergetic activation of obese ATMs, as the maximal OCR is actually reduced [196]. This observation suggests that additional mechanisms regulate ATM metabolism upon overnutrition. In addition to bioenergetic adaptations and proinflammatory activation, ATMs in obese WAT also trigger anti-inflammatory lipid metabolism programs. Those programs are driven by PPARγ (the master regulator of lipid catabolism) and p62 (a signaling-regulatory scaffold protein) and/or triggering receptor expressed on myeloid cells (TREM)-2 [185–187]. First, the PPARγ-target genes ATP binding cassette subfamily A member 1 (ABCA1, crucial for cholesterol export), CD36 (a scavenger receptor for lipid uptake) and/or perilipin 2 (PLIN2, a lipid droplet protein) are upregulated in omental and subcutaneous human ATMs from obese vs. nonobese individuals and in murine ATMs from obese WAT in concert with TNFα and IL-1β. Palmitate exposure also elevates PLIN2 and p62 expression in BMDMs. Blocking PPARγ and p62 in MMe-like activated BMDMs (treated with high levels of glucose, insulin, and palmitate) enhances IL-1β and/or TNFα expression. This observation illustrates the anti-inflammatory features of PPARγ/p62 and MMe-like activation, which is nevertheless distinct from M2-like activation [187]. Furthermore, a protective population of lipid-associated ATMs (LAMs) accumulated in CLS in mice and humans upon obesity. LAMs express TREM-2 and are characterized by transcriptional signatures of lysosomes/phagosomes, endocytosis, lipid metabolism (PPARγ) and OXPHOS. Although their functions have not been directly investigated, TREM-2 loss remodels ATM populations in obesity and enhances weight gain and adiposity [185]. In line with this, WAT ATMs are reduced in HFD-fed LysM-Cre PPARγ^f/f^ mice, but their adiposity and insulin and glucose tolerance are significantly enhanced [57].

In contrast, HFD-fed chimeric mice bearing bone marrow deficient in C/EBPβ, another regulator of lipid metabolism, also harbor reduced ATMs in WAT, but WAT inflammation (TNFα, IL-6, CCL2 and NLRP3) is also reduced. Despite the regulatory role of C/EBPβ in induced cytokine production [211], these results may suggest a context-dependent proinflammatory role of lipid accumulation in WAT ATMs [79]. Supporting this, proinflammatory CD11c^+^ ATMs in obese mice are known to express higher TNFα and IL-1β levels and accumulate more lipid droplets than anti-inflammatory CD11c^−^ ATMs [184, 192]. CD9^+^ proinflammatory ATMs are also lipid laden, accumulate in obese WAT and reside within CLS [183]. They display enhanced AP-1 and NF-κB activity and express proinflammatory gene signatures (TNFα, IL-1α, CCL2, IL-18) and lipid metabolism/lysosome gene signatures (PLIN2, CD63, LAMP2, LPL, LIPA). In addition, lipid-accumulating CD9^+^ ATMs have been found in the CLS of human WAT and are correlated with body mass. Notably, adoptive peritoneal transfer of obese fat-derived CD9^+^ ATMs into lean mice induces an inflammatory transcriptomic response in lean WAT highly reminiscent of the response in WAT upon overnutrition [183]. However puzzlingly, the described ATM populations appear to overlap; for example, TREM-2^+^ LAMs highly express CD9 [185], and the presence of ATMs is dynamic during obesity progression, with some populations increasing or decreasing over time.

However, the proposed functions of ATMs do not correlate with gross metabolic features, such as intracellular lipid accumulation. One explanation would be the nature/type of internalized lipids in ATMs, as full-length vs. truncated oxidized phospholipids or saturated vs. unsaturated fatty acids can induce opposing functions [189, 209].

Moreover, in addition to being diverse, ATMs may play different roles depending on the extent of obesity. Adipose tissue remodeling upon overnutrition is progressive and reversible upon HFD withdrawal. Despite increasing over time, the presence of (CD11c^+^) ATMs expressing proinflammatory genes (TNFα, IL-1β, IL-6) and lipid metabolism genes (CD36, PLIN2, ABCA1) and an inflammatory state in the WAT of HFD-fed mice actually precedes adipocyte death and CLS formation [184, 188]. The TLR2-myeloid differentiation factor (MYD)88-NADPH oxidase (NOX)2 axis controls both inflammation and lysosomal exocytosis, which is necessary for dead adipocyte clearance [208], in cultured MMe-like BMDMs. Consistently, NOX2^−/−^ mice display improved glucose tolerance correlated with decreased TNFα, IL-1β and IL-6 expression by WAT ATMs upon short-term HFD feeding. Conversely, at later time points, NOX2^−/−^ mice develop hepatosteatosis, insulin resistance and lipoatrophy associated with dead adipocyte accumulation and defective expression of lysosomal exocytosis genes by ATMs [188]. Moreover, upon switching of mice from a HFD to a normal chow diet, the numbers of proinflammatory CD11c^+^ ATMs are maintained in WAT, but TNFα and IL-1β expression in these cells is reduced, and insulin sensitivity is improved [184]. Hence, depending on the extent of WAT hypertrophy, the activities of ATMs may be beneficial or unfavorable, and lipid-laden ATMs can adapt their proinflammatory properties accordingly.

These factors should be considered when designing future studies to improve our understanding of how metabolism dictates ATM function, thus defining potential targets to combat overnutrition-associated pathologies.

## Macrophage metabolism in atherosclerosis

Atherosclerosis is a chronic inflammatory disease in which lipids build up in arteries, causing local inflammation and the development of atheroma plaques. These plaques, which are infiltrated by immune cells, can impede blood flow and eventually cause blood clots due to their rupture. In humans, macrophage plaque infiltration is related to altered plaque metabolism (increased glycolysis and hypoxia [212, 213]), necrotic core formation, enhanced plaque rupture and acute clinical cardiovascular events [214, 215]. Indeed, macrophages play a prominent role in atherosclerosis, influencing disease initiation, progression and regression [216].

At early stages, macrophages take up (modified) LDL that is retained in the arterial subendothelium, which leads to intracellular lipid accumulation and macrophage foam cell formation [217]. Macrophage foam cells have a diminished migratory capacity, which reduces their ability to egress plaques [218, 219], an important contributor to potential atherosclerosis regression, and show increased expression of lipid handling genes [220]. In plaques, macrophages can also display a proinflammatory phenotype that promotes the secretion of cytokines and chemokines and amplifies the immune response by recruiting monocytes, T cells and neutrophils [216].

As the lesion progresses, macrophages proliferate, release cytokines and proteases, and ultimately become apoptotic upon unresolved lipid and endoplasmic reticulum (ER) stress [216]. In advanced plaques, efferocytosis (phagocytosis of dead cells) is impaired, contributing to necrotic core formation. If dyslipidemia is resolved by transplanting the atherosclerotic aortic arches from apolipoprotein E (ApoE)^−/−^ mice into healthy recipients or via dietary changes in regression models, CD68^+^ plaque macrophage numbers decrease. In part, this is achieved by CC-chemokine receptor (CCR)7-mediated egress of macrophages from the plaques. The remaining plaque macrophages display transcriptional changes, including increased expression of the anti-inflammatory markers arginase-1 and CD163 [221, 222].

Analysis of the metabolic adaptations of atherosclerotic plaque macrophages is challenging due to the scarcity of these cells, and the existing knowledge is largely based on transcriptomics and the use of animal models with genetic deficiencies. Here, we outline current models of the metabolism of atheroma macrophages, which appear to be highly disease stage- or context-dependent and are often even opposing. This issue can additionally be explained by the fact that current knowledge on atherosclerotic macrophages is largely obtained by studies using BMDMs or peritoneal macrophages as surrogates. Given the functional and metabolic diversity of tissue macrophage types (Table 1) and BMDMs (outlined above), their resemblance may be incomplete.

### Lipid handling impacts macrophage function during hypercholesterolemia/atherosclerosis

Efferocytosis, macropinocytosis and phagocytosis together with diverse scavenger receptors (including CD36, SRA1/2, SRB1, SR-PSOX, LOX and LRP1) are involved in lipid uptake by macrophages [217]. In plaques, macrophages internalize environmental lipids such as LDLs, modified LDLs (mainly oxidized), oxidized phospholipids, fatty acids, and apoptotic cell-derived lipids (Fig. 2C).

Oxidized phospholipids are phosphocholine-containing phospholipids with polyunsaturated fatty acid moieties (mainly arachidonic acid) that are oxidized (e.g., by free radicals from the inflammatory environment) [223]. Oxidized phospholipids are recognized by CD36 and induce proatherogenic immune activation in peritoneal macrophages [224, 225]. In BMDMs, stimulation with oxidized phospholipid 1-palmitoyl-2-arachidonoyl-*sn*-glycero-3-phosphorylcholine (oxPAPC) and LPS drives simultaneous glycolysis and OXPHOS as well as glutamine catabolism and oxaloacetate accumulation. This metabolic reprogramming potentiates HIF-1 stabilization, which, in turn, increases hyperinflammation by promoting IL-1β production [226] (Fig. 2C). The oxPAPC-dependent genetic signature in mice is also upregulated in human individuals with pro-atherosclerotic lipid profiles [226].

As detailed earlier, the oxLDL/CD36 axis switches fatty acid metabolism and mitochondrial OXPHOS toward glycolysis, superoxide production and proinflammatory activity in peritoneal macrophages ex vivo [115] (Fig. 3B). Under atherogenic conditions in HFD-fed ApoE^−/−^ mice, increased GLUT1 expression and mitochondrial ROS levels are detectable in blood Ly6C^hi^ monocytes; the latter are also found in aortic lesional CD36-expressing F4/80^+^ macrophages [115]. Cholesterol crystals formed by CD36-mediated uptake of oxLDL by peritoneal macrophages activate the NLRP3 inflammasome [227], which is linked to Western diet-induced inflammation and aortic plaque formation in LDL receptor (LDLR)^−/−^ mice [228]. In this line, ApoE^−/−^ IL-1β^−/−^ mice show reduced aortic lesion sizes [227, 229], and anti-IL1β therapy has been found to lead to a lower rate of recurrent cardiovascular events in a human cohort independent of lipid-level lowering [230].

Cholesteryl esters of LDL are metabolized in the endolysosomal compartment into free cholesterol and fatty acids. Free cholesterol is either effluxed from cells to HDL (reverse cholesterol transport) via the lipid transporters ABCA1 and ABCG1, key players in atherosclerosis, or trafficked to the ER, where it is reconverted into cholesteryl esters and stored in lipid droplets [231] (Fig. 2C). As the lesion progresses, impairment of cholesterol trafficking in macrophages and the resulting accumulation of cytotoxic free cholesterol have been hypothesized to occur. In cultured peritoneal macrophages, cholesterol exposure activates the unfolded protein response, leading to ER stress and increased C/EBP homologous protein (CHOP)-mediated apoptosis [232]. Furthermore, cultured ABCA1^−/−^ and/or ABCG1^−/−^ macrophages increase their cellular cholesterol content (in lipid rafts), which activates TLR4 and NF‐κB signaling, resulting in the secretion of proinflammatory cytokines [233, 234] (Fig. 2C). LDLR^+/−^ mice that received ABCA1^−/−^ ABCG1^−/−^ bone marrow display enhanced foam cell presence and aortic lesion sizes [234], supporting the hypothesis that foamy macrophages are proinflammatory and drive atherosclerosis pathology.

In contrast, peritoneal macrophages of LDLR^−/−^ mice fed a high cholesterol/high fat (HCHF) diet increase their cholesterol content, leading to a subsequent decrease in the cholesterol biosynthesis pathway that leads to the accumulation of desmosterol, a cholesterol precursor that acts as a LXR ligand [235]. Desmosterol promotes LXR-mediated cholesterol efflux in peritoneal macrophages via LXR target genes such as ABC transporters, which is linked with attenuation of TLR signaling and decreased inflammatory signatures [233, 235, 236]. Decreased inflammation in conditions of hypercholesterolemia has also been shown to occur in peritoneal macrophages from LDLR^−/−^ mice fed an HCHF diet via suppression of the PPP and NRF2 oxidative stress pathways [22].

These discrepancies could be explained by the characterization of foamy and non-foamy macrophages that coexist in atherosclerotic plaques and exhibit different features [220]. Foamy macrophages in lesions show increased expression of OXPHOS-related genes as well as cholesterol metabolism, lysosome activity and PPAR signaling signatures. Conversely, non-foamy macrophages are enriched with genes involved in inflammatory processes, suggesting that these newly recruited macrophages promote local inflammation and plaque progression [220]. Hence, the differences between pro- and anti-inflammatory features exhibited by atherosclerotic plaque macrophages could be related to the coexistence of distinct macrophage types or states at different stages of disease.

Moreover, even the roles of individual factors involved in cholesterol handling by aortic plaque macrophages appear to be context dependent, such as in the case of LRP1, a marker usually linked with plaque progression under proatherogenic conditions [237]. First, peritoneal macrophages deficient in LRP1 display increased reverse cholesterol efflux, and ApoE^−/−^ mice grafted with LRP1^−/−^ bone marrow exhibit accelerated plaque regression [222]. These mice show reduced plaque CD68^+^ macrophage numbers and enhanced macrophage CCR7 expression, which contributes to atherosclerosis resolution in addition to the increased reverse cholesterol transport induced by LRP1 deficiency [222]. These two mechanisms may be intricately linked, as macrophage LXR signaling, a key pathway in cholesterol homeostasis, is required for maximal CCR7 expression and macrophage egress from plaques [238]. In contrast, a study using a LRP1^Y63F^ knock-in mouse model that carries a point mutation that impedes LRP1 phosphorylation and subsequent signal transduction has highlighted a role for LRP1 in promoting atherosclerosis [239]. Peritoneal macrophages from LDLR^−/−^ mice carrying LRP1^Y63F^ bone marrow show enhanced intracellular lipid accumulation and decreased clearance of apoptotic cells during disease progression due to a reduction in LXR- and ABCA1-mediated cholesterol efflux. Overall, the contradictory roles of the same pathway/receptor in atherosclerosis are possibly related to the different stages of atherosclerosis (regression vs. progression) [240].

Finally, in vitro studies in BMDMs, peritoneal macrophages and cell lines have shown cholesterol efflux to be controlled by microRNA (miR)-33, which affects several aspects of cellular metabolism, including FAO, mitochondrial respiration, gluconeogenesis and autophagy [241–244] (Fig. 2C). High miR-33a/b and low mitochondrial gene expression have been reported in human atherosclerotic plaques compared with healthy arteries, while other metabolic processes have not been assessed. Antagonism of miR-33 in ApoE^−/−^ or LDLR^−/−^ mice reduces aortic lesion size, lipid content, CD68^+^ plaque macrophage presence and ABCA1 expression, as determined after laser-capture microdissection [241, 245]. However, mitochondrially impaired PGC-1α^−/−^ peritoneal macrophages do not intrinsically alter their ATP levels but exhibit blunted miR-33 efficacy, suggesting additional mechanisms by which miR-33 controls ATP and cholesterol efflux [241]. Indeed, a steroidogenic acute regulatory protein (StAR)/mitochondrial sterol 27-hydroxylase (CYP27A1) axis also activates LXR-dependent cholesterol efflux and inflammatory gene expression in cultured macrophages and cell lines [246, 247]. In line with this, ApoE^−/−^ mice systemically overexpressing StAR due to infection with a recombinant CMV-StAR adenovirus exhibit decreased cholesterol and triglyceride accumulation in the liver as well as decreased aortic neutral lipid levels, although the relevance of these findings for macrophages have not been investigated [246].

Fatty acid accumulation can also promote a proinflammatory macrophage phenotype by inducing the production of fatty acid binding proteins (FABPs) in peritoneal macrophages [248, 249] (Fig. 2C). FABP4 deficiency attenuates inflammatory cytokine production in macrophage cultures [248], and FABP5 limits PPARγ activation and promotes Akt- and NF-κB-linked inflammation in peritoneal macrophages [249]. This results in reduced inflammation and decreased atherosclerosis in ApoE^−/−^ or LDLR^−/−^ mice transplanted with FABP4^−/−^ and FABP5^−/−^ bone marrow, respectively [248, 249]. In contrast, polyunsaturated fatty acids, mainly omega-3 fatty acids and arachidonic acid, can be converted through lipoxygenase pathways into pro-resolving lipid mediators such as lipoxins or resolvins that induce macrophage efferocytosis and anti-inflammatory gene expression [250] (Fig. 2C). Macrophages overexpressing 12/15-lipoxygenase (12/15-LO) show increased levels of lipoxin A4, which downregulates several proinflammatory cytokines, resulting in atheroprotection in ApoE^−/−^ mice overexpressing 12/15-LO in macrophages and increased atherosclerosis in ApoE^−/−^ 12/15-LO^−/−^ mice [251].

### Changes in glucose and amino acid metabolism in macrophages as atherosclerosis evolves

The atherosclerotic plaque microenvironment is characterized by hypoxic regions [252, 253] that drive metabolic reprogramming of cultured BMDMs and human blood monocytes toward increased glycolysis via the activation of HIF-1α, which in turn stimulates IL-1β production [254, 255]. In murine and human plaques, HIF-1α has been found to colocalize with CD68^+^ macrophages and highly expressed proteins involved in glucose metabolism, including GLUT1 and Hexokinase-2. High levels of IL-1β are also correlated with macrophage-rich regions [254, 255]. Cultured macrophages with stabilized HIF-1α in hypoxia increase triglyceride and sterol biosynthesis and halt cholesterol efflux via ABCA1 [254] (Fig. 2C), a potential mechanism by which plaque macrophages accumulate cholesterol. The inflammatory stimuli oxLDL and oxPAPC can also activate HIF-1α, stimulating glucose uptake and enhancing IL-1β production, respectively [115, 226]. HIF-1α^−/−^ bone marrow transplantation into LDLR^−/−^ mice results in decreased aortic lesion sizes, indicating the importance of HIF-1α in plaque macrophages [256].

In vitro studies have suggested that the effects of HIF-1α are due to its regulation of glucose uptake and glycolysis [256]; however, the role of glycolysis in plaque macrophages remains unclear. Bone marrow transplants overexpressing Glut1 increase macrophage glycolysis and PPP activation but do not alter inflammation or atheroma plaques in LDLR^−/−^ mice [257]. Similarly, LDLR^−/−^ mice with macrophage-specific deficiency of Glut1 show no difference in aortic lesion size but show an increased proportion of plaques with necrotic cores and decreased plaque stability [153, 258]. Moreover, an enhanced ^18^F-FDG signal, an indication of glucose uptake, corresponds to macrophage abundance and/or inflammatory activation in atherosclerotic plaques in both humans [212] and rabbits [259]. Of note, compared with healthy control monocytes, ex vivo LPS/IFNγ-stimulated circulating monocytes from atherosclerosis patients show increased glycolysis and PPP gene expression as well as enhanced IL-1β and IL-6 production via the ROS/pyruvate kinase M2/STAT3 pathway [260].

Amino acids in the microenvironment can also play roles in macrophage metabolism and function in atherosclerosis. For instance, the increased systemic availability of leucine upon high-protein diet feeding is associated with increased atherosclerotic plaque complexity via alteration of the mTORC1/autophagy axis in peritoneal macrophages [261]. Macrophage culture experiments have shown that leucine first synergizes with proatherogenic lipids (i.e., 7-ketocholesterol and cholesterol crystals) to induce mitochondrial uncoupling and increase macrophage ROS production. Then, mTORC1 is activated, which inhibits the mitophagy of dysfunctional mitochondria. These leucine-induced mechanisms trigger macrophage apoptosis in plaques, which in turn contributes to necrotic core formation and increases plaque instability upon high-protein diet feeding [261].

Moreover, oxPAPC-triggered hyperinflammation in BMDMs is dependent on glutamine and ATP citrate lyase (ACLY)-dependent conversion of citrate into oxaloacetate. Indeed, the reductions in atherosclerotic plaques in LDLR^−/−^ mice upon treatment with glutaminase or ACLY inhibitors as well as in LDLR^−/−^ mice harboring LysM-Cre ACLY^f/f^ bone marrow support the importance of glutamine catabolism and oxaloacetate accumulation in atherosclerosis [226, 262].

In summary, different types of metabolites (lipids, glucose, and amino acids), lipid handling processes and the involved signaling factors have been shown to foster diverse features in atheroma macrophages during disease progression or regression. This controversy highlights the need for accurate evaluation of the specific microenvironmental conditions (i.e., lipid type/source, stage of the disease, hypoxic regions) [263] as well as of the model system used (i.e., ex vivo vs. in vivo, peritoneal and/or aortic macrophages) [179] when investigating complex diseases such as atherosclerosis.

## The metabolic challenges of macrophages in cancer

### Overview of the tropic functions of tumor-associated macrophages and underlying metabolic features

Solid tumors form complex structures with requirements similar to those of developing organs [264]. Tumors recruit circulating monocytes into the tumor microenvironment (TME), where they become tumor-associated macrophages (TAMs) [265]. However, TAMs are not only of monocytic origin but can also be derived from tissue-resident embryo-derived macrophages [266, 267]. Depending on the tumor type, the pro- or anti-tumorigenic roles of monocytes or embryo-derived macrophages vary [268, 269], and the field of tumor macrophage ontogeny is in its infancy.

Generally, a high TAM content correlates with a poor prognosis [270, 271], and TAMs are associated with anti-inflammatory properties as well as a broad spectrum of metabolic features [272]. Notably, anti-inflammatory TAMs strongly rely on glycolysis, in contrast to cultured BMDMs (Fig. 1A), demonstrating the complexity of in vivo macrophage polarization [273, 274]. TAM heterogeneity also extends to different tumor nodules of the same animal [275], as the TAM phenotype changes over time depending on tumor progression. The ways in which TAMs support tumor development are also diverse, such as generation of an immunosuppressive TME by anti-inflammatory cytokines and programmed death-ligand 1 (PD-L1) expression [276, 277], extracellular matrix remodeling [278] and facilitation of the angiogenic switch for large tumor nodules [279]. However, macrophages are not always tumorigenic [280]. Increased macrophage infiltration prolongs survival in cervical carcinoma patients and is associated with mature CD163^-^ proinflammatory macrophages that promote cytotoxic CD8^+^ T cell responses [281–283]. In colorectal cancer, an improved prognosis also correlates with macrophage infiltration, regardless of phenotype (based on nitric oxide synthase 2 [NOS2] or CD163 expression) [284, 285].

One of the main metabolic pathways in macrophages shown to influence tumor growth is amino acid metabolism, and protumoral TAMs frequently highly express arginase-1 [14, 286] (Fig. 4.1). Tumor-associated myeloid cells express cationic amino acid transporters 1 and 2B at higher levels than nontumor-associated myeloid cells, leading to higher uptake of arginine and its depletion in the TME [287, 288]. The result of this is three-fold: First, arginase-1 converts arginine into ornithine and urea, inhibiting tumoricidal NO synthesis. Second, arginine is metabolized into ornithine and polyamines, which support tumor growth. Myeloid-specific deletion of ornithine decarboxylase, a rate-limiting factor in the polyamine biosynthesis pathway, also leads to increased production of M1-associated cytokines, including TNFα, IL-1β, IFNγ, and NOS2, resulting in decreased tumor burden and improved survival in a model of colitis-associated carcinogenesis [289]. Third, the depletion of arginine from the TME suppresses the anti-tumoral activity of T cells [286–288, 290].

**Fig. 4:**
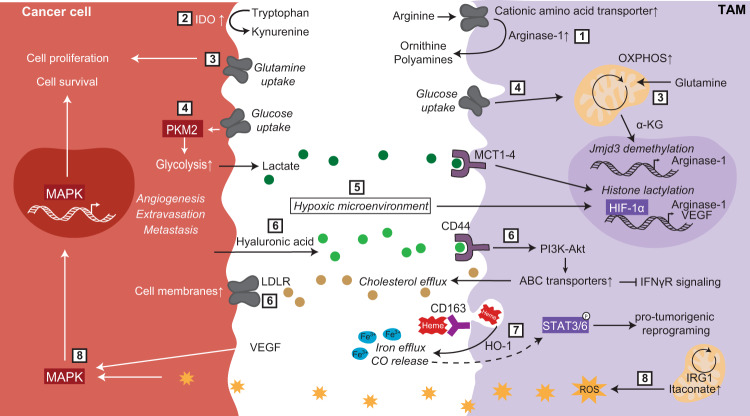
Tumor-associated macrophage and cancer cell cross-talk. Cancer cells and tumor-associated macrophages engage in metabolic cross-talk, which has been shown primarily to support tumor cell growth and survival. This includes increased arginase expression by macrophages promoting arginine metabolism (1); tryptophan metabolism in cancer cells via IDO (2); glutamine metabolism in cancer cells to promote their proliferation and in macrophages to drive anti-inflammatory gene expression (3); glucose metabolism-mediated increases in extracellular lactate, which modulates macrophage function (4); the induction of and response to a hypoxic microenvironment (5); cholesterol export from macrophages (via ABC transporters) to cancer cells (via LDLR) (6); heme metabolism and iron efflux from macrophages (7); and mitochondrial ROS production (8). More details of these pathways can be found in the main text. CO, carbon monoxide. Solid lines: direct relationships; dashed lines: indirect relationships. Brown circles: bound cholesterol/LDL/oxLDL; dark green circles: lactate; light green circles: hyaluronic acid; orange stars: ROS

Tryptophan metabolism is also implicated in modulating anti-tumor immune responses (Fig. 4.2). The enzyme indoleamine-2,3-dioxygenase (IDO) performs the first step of the kynurenine pathway, which converts tryptophan to N-formylkynurenine. In the context of tumors, this has been linked to T cell suppression via tryptophan depletion from the TME and promotion of regulatory T cell responses [291]. However, IDO-overexpressing tumors also recruit more TAMs into the TME, where they express CD206 and high levels of TGFβ but low levels of NOS2, CD86, and IL-12—characteristics of protumorigenic macrophages [291]. This macrophage modulation appears to be dependent on IDO-induced macrophage expression of the arylhydrocarbon receptor [291, 292]. Arylhydrocarbon receptor signaling also induces the expression of cytochrome p450 enzymes, such as CYP1A1, which are involved in steroid and lipid metabolism [293], yet their roles in cell-intrinsic TAM metabolism require further study.

Glutamine metabolism is known to fuel biosynthesis pathways in cancer cells, contributing to cell survival; however, this pathway may also directly contribute to TAM function [294] (Fig. 4.3). In cultured BMDMs, glutamine drives M2 macrophage polarization via epigenetic modifications such as Jmjd3 demethylation that enhance the expression of key M2 genes, such as arginase-1, and UDP-GlcNAc synthesis [25, 56]. Supporting these findings, genetic deletion of glutamine synthase in macrophages leads to more M1-like features, as defined by MHCII^hi^ expression, and reduces the presence of CD206^+^ M2 macrophages. This strategy does not affect the primary tumor burden; however, it does inhibit metastasis and increase T cell infiltration [295].

### How are TAMs metabolically repurposed to be protumorigenic?

TAMs and tumor cells continuously interact, similar to macrophages and their surrounding parenchyma in homeostasis [296]. For example, tumor-produced M-CSF stimulates TAM production of epidermal and vascular endothelial growth factor (EGF and VEGF), which activate tumor cells in a paracrine loop to promote extravasation and enhance metastasis [279, 297, 298]. Tumor cells and TAMs also engage in metabolic crosstalk. Tumor cells are typically highly glycolytic due to exclusive expression of the M2 isoform of pyruvate kinase (PKM2) [39, 299], which can lead to competition for nutrients with other glycolytic cells in the TME [300]. TAMs have the highest level of glucose uptake, followed by T cells and then tumor cells, all of which are dependent on mTORC1 activity [301]. In contrast, tumor cells have the highest uptake of glutamine, indicating that glycolytic tumors may not be immunosuppressive through direct nutrient competition but via more complex intercellular regulation [301]. The end-product of tumor cell glycolysis is lactate, which promotes the production of VEGF and arginase-1 in TAMs to support tumor vascularization and proliferation (Fig. 4.4). Indeed, tumor cells genetically modified to produce less lactic acid show reduced growth in vivo. This effect is dependent on lactic acid uptake via monocarboxylate transporters (MCT1-4) and macrophage expression of HIF-1α [150]. This finding is supported in vitro, where lactate induces anti-inflammatory gene expression programs, e.g., an increase in arginase-1 and a decrease in iNOS, in BMDMs through histone lactylation [302]. Alongside lactate, the hypoxic TME also induces HIF-1α stabilization in TAMs, which again triggers VEGF and arginase-1 expression [150] (Fig. 4.5). In line with this, analysis of TAM heterogeneity has shown denser localization of anti-inflammatory macrophages in more hypoxic areas of a mammary adenocarcinoma model [303].

Tumor-derived succinate also promotes succinate receptor 1 (SUCNR1)/phosphoinositide 3-kinase (PI3K)/HIF-1α-dependent polarization of peritoneal macrophages in vitro, enhancing macrophage expression of arginase-1 [304]. Apart from modulating the TME, tumors can actively export exosomes that alter TAM functions. The microRNA let-7a, found in tumor exosomes, inhibits mTOR signaling in TAMs, leading to increased OXPHOS and expression of the M2-like markers CD206 and arginase-1 [305]. It is not clear whether protumoral macrophage polarization occurs in newly recruited macrophages or upon repolarization of the proinflammatory phenotype of existing TAMs, as reversion toward an anti-tumoral TAM phenotype appears to be limited [46].

Lipid handling by macrophages in the TME is also key to determining cancer outcomes. ABCG1-deficient macrophages, with reduce cholesterol efflux, improve outcomes in bladder carcinoma and melanoma models due to a shift to M1-like gene expression—increasing iNOS, MHCII, and CD86 expression while decreasing arginase-1 and CCL22 [306]. Similarly, in vitro, ABCG1-deficient macrophages show defective lipid efflux, accumulate cholesterol in lipid rafts and enhance proinflammatory cytokine production [307, 308]. The secretion of hyaluronic acid by ovarian cancer cells, an occurrence observed in many cancers, promotes plasma membrane cholesterol efflux in TAMs via binding of hyaluronic acid to CD44 [309] (Fig. 4.6). This reduction in intracellular cholesterol enhances IL-4 receptor (IL-4R)/STAT6/PI3K signaling in vitro and in vivo, promoting the expression of the typical M2 marker arginase-1 while inhibiting NOS2 and proinflammatory IL-12 expression, thereby promoting tumor progression [309, 310]. This cholesterol efflux is dependent on PI3K and Akt activation, which can upregulate ABCA1 expression in macrophages [309, 311]. Moreover, cholesterol efflux leads to decreases in lipid rafts in macrophages, which act as signaling platforms for various receptors, including IFNγR, decreasing IFNγ-dependent expression of the proinflammatory genes IL-12 and iNOS [309]. Indeed, myeloid-specific deletion of cholesterol efflux genes ABCA1 and ABCG1 or inhibition of IL-4R signaling, STAT6, or PI3K has been found to significantly impair tumor progression in an ovarian cancer model [309]. Moreover, cancer cells upregulate cholesterol receptors to utilize the cholesterol lost from TAMs [312].

In addition to promoting cholesterol efflux, PI3K signaling regulates many key genes for immune activation or suppression in the TME [313, 314]. However, lipid handling requires a delicate balance, as lipid accumulation in RXRα-deficient macrophages can lead to apoptosis in the cancer setting. Peritoneal LPMs are the main infiltrating immune cells in ovarian cancer, where they support tumor growth, and their lipid-induced apoptosis decreases tumor growth [119]. In another context, compared with healthy control cells, TAMs from the bone marrow of patients with myeloma display increased CD36-mediated lipid uptake and storage [315]. This finding correlates with an increased FAO transcriptional signature and mitochondrial respiration in TAMs and supports the protumoral functions of these cells via the expression of arginase-1, VEGF, HIF-1α, and CCL2 [315].

Increased pyrimidine synthesis and release by protumoral macrophages also nurtures tumor cells [316]. Similarly, while proinflammatory macrophages typically sequester iron to limit pathogen growth, protumoral TAMs increase iron export, which is hijacked by tumors to promote cell proliferation and tissue repair [317]. A distinct set of bone marrow-derived F4/80^hi^ TAMs with high expression of the hemoglobin scavenger receptor CD163 and the heme catabolism enzyme HO-1 accumulate at the invasive margins of tumors [318] (Fig. 4.7). These TAMs rely on an NF-κB1/M-CSF receptor/C3a anaphylatoxin receptor signaling axis to drive HO-1 expression. The effect of heme catabolism is to remove cytotoxic labile heme from the TME and release carbon monoxide, which enhances STAT3/STAT6 activation in TAMs to induce anti-inflammatory cytokines such as IL-10 (Fig. 4.7). In a myeloid-specific HO-1-deficient mouse model, TAM heme catabolism was found to have no effect on primary tumors but to support metastasis by promoting angiogenesis and epithelial-to-mesenchymal transition [318].

ROS and reactive nitrogen species (RNS) are produced by macrophages to kill pathogens, but their range of other functions can be both pro- and anti-tumorigenic [319]. Macrophage-derived NO can directly induce tumor cell apoptosis [320]. However, TAM-derived ROS can also activate mitogen-activated protein kinase (MAPK)/PPARy signaling by tumors, promoting their survival (Fig. 4.8). TAMs are also enriched with antioxidant transcriptional signatures that are correlated with anti-tumorigenic responses [321]. In the context of peritoneal grafted cancer models, increases in FAO, OXPHOS, and glycolysis are observed in peritoneal resident macrophages [322]. IRG1 and itaconate are highly upregulated in these macrophages, resulting in increased OCR and mtROS production, which activates MAPK in nearby tumor cells to enhance their survival [322] (Fig. 4.8). Certain tumors can also induce ROS accumulation to support their own growth and influence other cells in their microenvironment, including TAMs [323]. Indeed, in a carcinogen-induced lung cancer model, administration of the ROS inhibitor butylated hydroxyanisole (BHA) prevented TAM recruitment into the TME and subsequently decreased tumor burden [324].

Finally, while TAMs are promising therapeutic targets, the current knowledge suggests that their targeting should be context dependent. Apart from clinical strategies reducing TAM presence, repurposing of TAMs from an anti-inflammatory to an anti-tumor phenotype is being pursued [179, 325]. For instance, CpG oligodeoxynucleotides, which are TLR9 agonists, induce a unique type of metabolic rewiring in macrophages whereby they do not utilize exogenous fatty acids but incorporate glucose into acetyl-CoA to enhance de novo lipid biosynthesis for FAO and membrane expansion [326]. This metabolic change increases membrane fluidity and subsequently phagocytic activity, enhancing TAM anti-tumor functions in a model of pancreatic ductal adenocarcinoma [326]. Similarly, macrophages that utilize FAO appear to increase efferocytosis via PPAR activation [327]. However, a better understanding of the unique metabolism of TAMs will improve future therapeutic approaches.

### Concluding remarks

Early in vitro studies associated the metabolic states of macrophages with functional features adopted upon activation (Fig. 1A). Indeed, parallels of this metabolic and functional polarization are observed during tissue injury, wound healing and regeneration. Macrophages initially adopt a proinflammatory and glycolytic state before switching to a resolving state and predominantly mitochondria-driven metabolism (Fig. 1B).

However, the homeostatic metabolic activities of tissue macrophage populations appear to be as diverse as their identities, the variety of microenvironments they reside in and, subsequently, the functions they execute to maintain homeostasis (Table 1). For example, osteoclasts specialize in bone matrix degradation, which is driven by glycolysis, while splenic RPMs engage in iron recycling when phagocytosing defective erythrocytes, and peritoneal LPMs require active mitochondria to generate a respiratory burst upon encountering yeast (Fig. 3). In addition, numerous tissue macrophage populations, such as pulmonary AMs, splenic RPMs, hepatic KCs, and peritoneal LPMs, express the metabolic machinery for lipid and cholesterol handling to clear environmental factors (Fig. 2A). Anatomical tissue niches for resident macrophages thus direct tissue macrophage identity and contribute to the metabolic features the macrophages adopt. For example, epithelial or stromal cell-derived signaling factors such as GM-CSF or Notch ligands induce PPARγ in AMs or Spi-C and LXRα expression in KCs, respectively. Additionally, metabolites present in “soluble” tissue niches foster metabolic traits of tissue macrophages, such as splenic heme for RPMs and glutamate for LPMs.

In line with this, environmental changes accompanying or underlying certain pathologies can overwhelm or hijack tissue macrophages that try to maintain their homeostatic activity. The resulting metabolic adaptations of tissue macrophages not only alter macrophage functionality but can also contribute to or drive disease severity. Indeed, adipose tissue macrophages during overnutrition-mediated WAT hypertrophy or plaque macrophages during atherosclerosis can be overpowered in their attempts to clear the accumulating extracellular lipid load, which culminates in proinflammatory responses (Fig. 2B, C). Tumor-associated macrophages also react functionally with underlying metabolic changes to cues present in cancer microenvironments and actively engage in metabolic crosstalk with cancer (and other) cells (Fig. 4), which often fosters cancer progression. Despite these emerging profound changes in tissue macrophage metabolism that underlie their functionality in pathologic settings, this avenue has been explored surprisingly little as a therapeutic option in the clinic [179]. Currently, efforts predominantly aim to target macrophages [180, 328, 329] or metabolic features locally/systemically [156–158, 173, 330, 331] in distinct pathophysiological settings. A joint effort of these approaches to affect metabolism specifically in macrophages is needed and may hold great therapeutic potential and limit side effects.

To facilitate translational application, we have to improve our knowledge of tissue macrophage metabolism in homeostasis and disease, since related reports often extrapolate observations from cultured surrogates, which themselves represent cells under metabolic challenge, to in vivo tissue macrophages. While AMs and LPMs can be harvested without tissue dissociation, the purification of macrophages located within the tissue stroma is more complex and can alter the metabolic profiles of the macrophages. Nevertheless, for some mainly murine macrophage populations, very informative techniques such as bulk transcriptomics and metabolomics have been performed, and single-cell-based analyses of murine and human tissues are emerging [332]. Met-Flow and SCENITH, flow cytometry-based methods using specific inhibitors to dissect functional (energetic) metabolic profiles at a single-cell level, represent feasible alternatives for complex tissues/samples [333, 334]. CyTOF panels with large numbers of key metabolic enzymes have been successfully used in recent years to study metabolic changes [335]. These cytometry-based techniques have also been applied to human myeloid cells in blood or TAMs. Spectroscopy and fluorescence imaging techniques using ratiometric fluorescence probes that detect changes in metabolic features [126, 336] are emerging as valuable tools to study tissue macrophage metabolism as well as enzyme histochemistry and histocytometry for measurement of enzyme activity. Finally, whole-body imaging systems such as positron emission tomography, single-photon emission computed tomography or nuclear magnetic resonance have also been successfully applied [12].

In addition, genetically modified animal models represent valuable tools for the investigation of tissue macrophage metabolism. Cre recombinase-based driver lines, such as LysM-Cre lines, and/or bone marrow replacement strategies have been successfully used to dissect the effects of metabolic alterations in macrophages in vivo. However, apart from a few exceptions [108, 337], these approaches are mostly not specific for particular tissue macrophage subsets or even macrophages, which might limit the interpretation of the results. The fact that bona fide macrophages are often rapidly out-populated by bone marrow-derived macrophages in tissues in many disease settings (the macrophage disappearance reaction) [110] can further influence the complexity of studying tissue macrophage metabolism. Moreover, some features related to cellular metabolism have been found to be distinctly regulated in murine and human macrophages, such as iNOS and arginase expression [338]. In addition, the dependence of the bioenergetics of monocytes on glucose or mitochondrial metabolism differs between humans and mice [333]. These observations clearly highlight the need for the validation of findings from mouse model systems in the human setting to be clinically relevant.

Overall, in light of the emerging importance of the microenvironment for cellular metabolism (Figs. 2–4), a complementary combination of the currently available techniques applied to tissue macrophages in vivo appears most suitable for reliable investigation of the metabolic traits, dependencies and adaptations of these cells. Understanding the metabolic features of tissue macrophages will provide valuable information for the maintenance of tissue homeostasis and likely help to control pathologies that are driven by metabolic changes in macrophages.
